# Phylogeography of *Nanorana parkeri* (Anura: Ranidae) and multiple refugia on the Tibetan Plateau revealed by mitochondrial and nuclear DNA

**DOI:** 10.1038/srep09857

**Published:** 2015-05-18

**Authors:** Jun Liu, Cuimin Wang, Dongli Fu, Xiaoju Hu, Xiangmo Xie, Pengfei Liu, Qiong Zhang, Meng-Hua Li

**Affiliations:** 1 CAS Key Laboratory of Animal Ecology and Conservation Biology, Institute of Zoology, Chinese Academy of Sciences (CAS), Beijing 100101, China; 2 Deep-Sea Research Department, Sanya Institute of Deep-Sea Science and Engineering, Chinese Academy of Sciences (CAS), Sanya 572000, China; 3 College of Life Sciences, University of the Academy of Sciences, Beijing 100049, China; 4 Gansu Provincial Key Laboratory of Herbivorous Animal Biotechnology, Gansu Agricultural University, Lanzhou 730070, China; 5 College of Life Sciences, Yangtze University, Jingzhou 434025, Hubei, China

## Abstract

Quaternary climatic changes have been recognized to influence the distribution patterns and evolutionary histories of extant organisms, but their effects on alpine species are not well understood. To investigate the Pleistocene climatic oscillations on the genetic structure of amphibians, we sequenced one mitochondrial and three nuclear DNA fragments in *Nanorana parkeri*, a frog endemic to the Tibetan Plateau, across its distribution range in the southern plateau. Mitochondrial cytochrome *b* (Cyt*b*) and three nuclear genes (*c-Myc2*, *Rhod*, and *Tyr*) revealed two distinct lineages (i.e. the lineages East and West), which were strongly geographically structured. The split of the two divergent lineages was dated back earlier than the Middle Pleistocene, probably being associated with climatic and ecological factors. Species distribution modeling, together with the phylogeographic structuring, supported the hypothesis of multiple refugia for *N. parkeri* on the Tibetan Plateau during the Pleistocene glaciations, and suggested the Yarlung Zangbo valley and the Kyichu catchment to be the potential refugia. Our findings indicate that Pleistocene climatic changes have had a great impact on the evolution and demographic history of *N. parkeri*. Our study has important implications for conservation of this and other frog species in the Tibetan Plateau.

Quaternary climatic oscillations greatly influenced the current distribution patterns and evolutionary histories of many organisms, which could facilitate population divergence and consequent speciation^1^,^2^. In particular, effects of the late Pleistocene climatic changes on phylogeographic structures in different species have been extensively investigated in Europe and North America^2^. However, these issues have not been as well understood in mountainous areas as in the continental regions^3^,^4^, especially in Asian high mountain ranges. Moreover, asynchronous glaciations appeared to occur between mountain-plateau and continental regions, and even within mountain-plateau regions^5^,^6^,^7^,^8^. In this context, the Tibetan Plateau is an ideal site to investigate the effects of climatic changes on genetic divergence and differentiation of alpine organisms.

The Tibetan Plateau is the highest, largest and one of the youngest plateaus on Earth, with an area of 2.5 million km^2^ and an average elevation of 4500 meters above sea level (m.a.s.l.). It comprises broad planation surfaces and basins in its central part, and most of the highest mountains along the margins of the plateau^9^. The uplift of the Tibetan Plateau has dramatically changed the topography of central Asia, probably being associated with the Quaternary glacial climate changes on this highland area^10^,^11^,^12^. Increasing evidence indicated that expanded ice caps and extensive valley glacier systems rather than an extensive ice sheet covered most of the Tibetan Plateau during the Quaternary glaciations^8^,^9^,^13^,^14^.

Effects of Pleistocene climatic changes on the distribution and evolution have been investigated in many species endemic to the Tibetan Plateau and surrounding regions^15^,^16^,^17^,^18^,^19^,^20^,^21^. Studies from different species at different parts of the Tibetan Plateau gave conflicting results. Some earlier studies suggested that the low-altitude periphery of the Tibetan Plateau (i.e. southeast or east region) served as glacial refugia for the flora and fauna living in the Tibetan Plateau and surrounding areas^15^,^16^,^18^,^22^,^23^. However, an increasing number of studies indicated multiple refugia on the plateau platform^17^,^24^,^25^. Therefore, more empirical data from additional endemic organisms are needed to thoroughly understand the effects of these historical events. Besides, most organisms investigated in previous studies occur on the edges of the Tibetan Plateau^15^,^21^,^23^. Only relatively few studies focused on the heterogeneous south and the interior region of the plateau, most of which focused on the alpine flora^16^,^17^,^19^,^24^.

Amphibian species are sensitive to environmental changes because of their physiological constraints, and therefore are regarded as good indicators of geological and climatic changes over time^26^,^27^,^28^. Among the anurans endemic to the Tibetan Plateau, *Nanorana parkeri* occupies an extensive altitudinal range from 2850 to 4700 m.a.s.l.^29^, making it the ranid species with the highest altitudinal distribution in the world^30^. This frog is widely distributed in the southern TP (28–31°N, 84–97°E), and is the only anuran species extending to the middle Yarlung Zangbo River of the interior plateau^29^. The southern TP is classified as an important biodiversity hotspot^31^, where the topography varies greatly, comprising complex drainage systems and most of the highest mountains in the world (Supplementary Fig. S1 online). The extreme landscape heterogeneity and high endemic species diversity in the region were believed to be profoundly influenced by the uplift of the Tibetan Plateau and/or the Quaternary climatic oscillations^17^,^19^,^24^.

In this study, we investigated the phylogeographic pattern and genetic population structure of *N. parkeri* in the Tibetan Plateau by analyzing the sequences of one mitochondrial (cytochrome *b*, Cyt*b*) and three nuclear DNA (cellular myelocytomatosis intron 2, *c-Myc2*; rhodopsin, *Rhod*; tyrosinase, *Tyr*) fragments. By collecting samples of *N. parkeri* across its range in the Tibetan Plateau, we aimed to (i) explore the genetic diversity and phylogeographic structure of the *N. parkeri* populations; (ii) examine whether the focal species survived in the plateau platform during the Pleistocene glaciations, or followed the pattern of glacial retreat to the low-altitude periphery and recolonization through postglacial expansion. The combination of phylogeographic analyses and species distribution modeling was used, which allow us to obtain a comprehensive picture of the phylogeography and historical demography of *N. parkeri*.

## Results

### Phylogeographic structure

**Figure 1:**
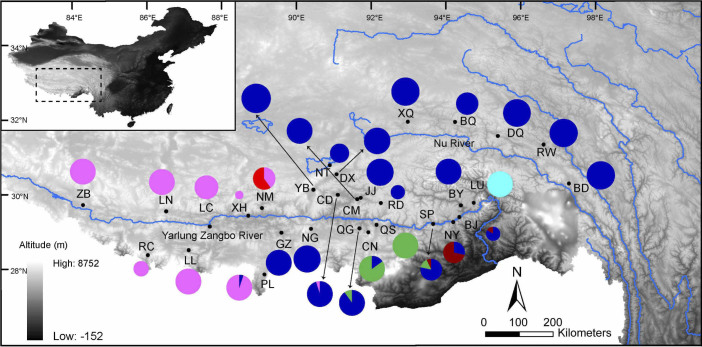
Map of sampling locations for *Nanorana parkeri*. The pies represent the Cyt*b* sublineage (E1–E4 and W1–W2) frequency in each population. Different colours correspond to different Cyt*b* sublineages in Fig. 2. Abbreviations of populations are detailed in Table 1. Haplotypes of each lineage in each population are shown in Supplementary Table S2 online. The map was drawn using ArcGIS v10.1 (ESRI, CA, USA) and Adobe Illustrator CS5 v15.0.0 (Adobe Systems Inc., San Francisco, CA).

A total of 533 specimens of *N. parkeri* from 30 localities (Fig. 1 and Table 1) were sampled on the Tibetan Plateau, generally representing the entire species distribution at present. Direct sequencing yielded 533 Cyt*b* sequences, among which 75 haplotypes were determined. A total of 268 sites were variable with 76 sites parsimony-informative. Sequences of nuclear gene (*c-Myc2*, *Rhod* and *Tyr*) segments were obtained from 530, 531 and 530 samples, respectively. A total of 24 haplotypes were identified in *c-Myc2*, and 12 sites were variable with seven parsimony-informative. Two haplotypes were found in *Rhod* with one parsimony-informative site. In *Tyr*, five haplotypes were identified, and four sites were variable with one parsimony-informative site. All the haplotypes were submitted to GenBank with accession numbers KJ434188-KJ434292 and KJ810612 (Supplementary Table S1 online).

**Figure 2:**
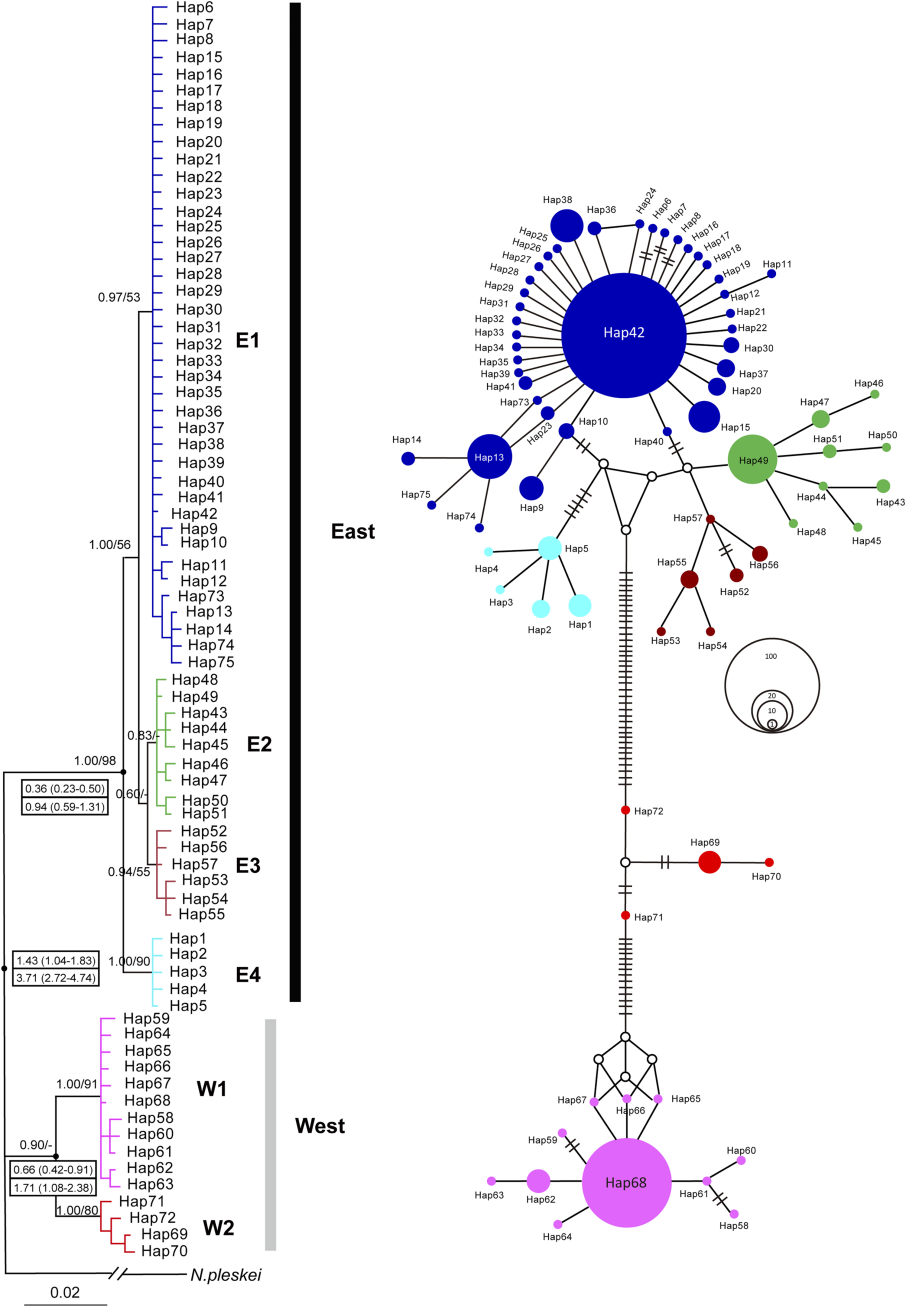
Bayesian phylogenetic tree (left) and the median-joining network (right) of mitochondrial Cyt*b* haplotypes for *N. parkeri*. Bayesian posterior probabilities/maximum likelihood bootstrap support values are above the branches. Mean time to the most recent common ancestor (TMRCA) with 95% highest posterior density (95% HPD) in parenthesis for the key nodes are given in the boxes (Mya): the above values are derived from the higher mutation rate (1.8 × 10^-8^/site/year) and the low from the mutation rate of 6.9 × 10^-9^/site/year. Colours in the network represent different Cyt*b* lineages. In the network, sizes of cycles indicate the haplotype frequencies. Network branches linking the cycles indicate one mutation step; two or more mutations are represented by slashes crossed with the network branches. The small open circles are hypothetical missing intermediates.

The phylogenetic relationships deduced from Cyt*b* sequences were identical using Bayesian inference (BI) and maximum likelihood (ML) approaches. Two main lineages (East and West) were identified, and the lineage East was strongly supported (Bayesian posterior probabilities/maximum likelihood bootstrap proportions: 1.00/98; Fig. 2). The two lineages comprised four (E1–E4) and two (W1 and W2) sublineages, respectively. Among them, sublineages E1, E4, W1 and W2 were highly supported by the Bayesian posterior probabilities (≥ 0.97). Mean pairwise uncorrected *p*-distances within each sublineages ranged from 0.2% to 0.3%. Distances between E1–E4 were 0.44 – 0.97%, while a much higher *p*-distance (1.96%) was observed between W1 and W2. Divergence between lineages East and West ranged from 4.24% to 4.35% (Supplementary Table S3 online).

Strong phylogeographic structure was observed for *N. parkeri* within its range (Fig. 1). All the individuals (excluding one from the population PL) belonging to the Cyt*b* lineage East (i.e. E1–E4) occupied the eastern region of the distribution area, while all the individuals (excluding one from CD) from the lineage West (i.e. W1 and W2) were confined to the western region. Sublineages E1 and W1 were most prevalent, while E2, E3, E4 and W2 occurred mostly in one or several populations. E2 was detected in four neighbouring populations (QG, CN, QS and SP). Similarly, E3 was restricted to three neighbouring populations (SP, NY and BJ). All individuals from the population LU belonged to E4, and W2 was only found in the population NM.

### Population genetic structure

The Cyt*b* haplotype diversity (*h*) was moderate to high (*h* = 0.52–0.85) in five of the six Cyt*b* sublineages (except W1), while the nucleotide diversity was quite low (*π* = 0.00080–0.00182; Table 2). Ten populations showed moderate to high haplotype diversity (*h* = 0.52–0.89; Supplementary Table S2 online), and eight of the 10 populations were distributed along the Yarlung Zangbo River. However, a very low level of nucleotide diversity was observed across all the populations (*π* = 0.00009–0.01109; Supplementary Table S2 online). Five populations consisted of only a single Cyt*b* haplotype (Hap68: LN and XH; and Hap42: NT, RD and XQ).

Pairwise Φ_ST _values ranged from 0.69 to 0.98 between the six Cyt*b* sublineages, and all the values were significant (*P* < 0.01; Table2). The AMOVA results showed strong genetic differentiation of *Cytb* sequences, with 93.2% of the total variance detected between the eastern and western regions (Table 3). Genetic divergence between the six Cyt*b* sublineages was also reflected in the Cyt*b* network (Fig. 2). Sublineages E1 and W1 showed star-like shapes, indicating recent population expansions. The dominant haplotype Hap42 of E1 occurred in 232 samples, and was distributed in 19 of the 22 eastern populations (except the populations LU, QS and YB; Supplementary Table S2 online). Similarly, the dominant haplotype Hap68 of W1 was found in all eight western populations, occurring in 95 samples. Surprisingly, 69 of the 75 (92%) haplotypes occurred in single populations (i.e. private haplotype). In addition to the two dominant haplotypes, only four haplotypes (Hap20, Hap36, Hap49 and Hap52) were shared between populations (Supplementary Table S2 online), and three pairs of populations were located near each other (i.e. populations CM and JJ for Hap36; CN and QS for Hap49; NY and SP for Hap52; Fig. 1). In addition, the haplotype network was characterized by a few missing intermediate haplotypes. These missing haplotypes might be extant but not sampled, or extinct. Given the extensive sampling and the heterogeneous landscape, this observation implies that some populations carrying the intermediate haplotypes most probably have become extinct.

**Figure 3:**
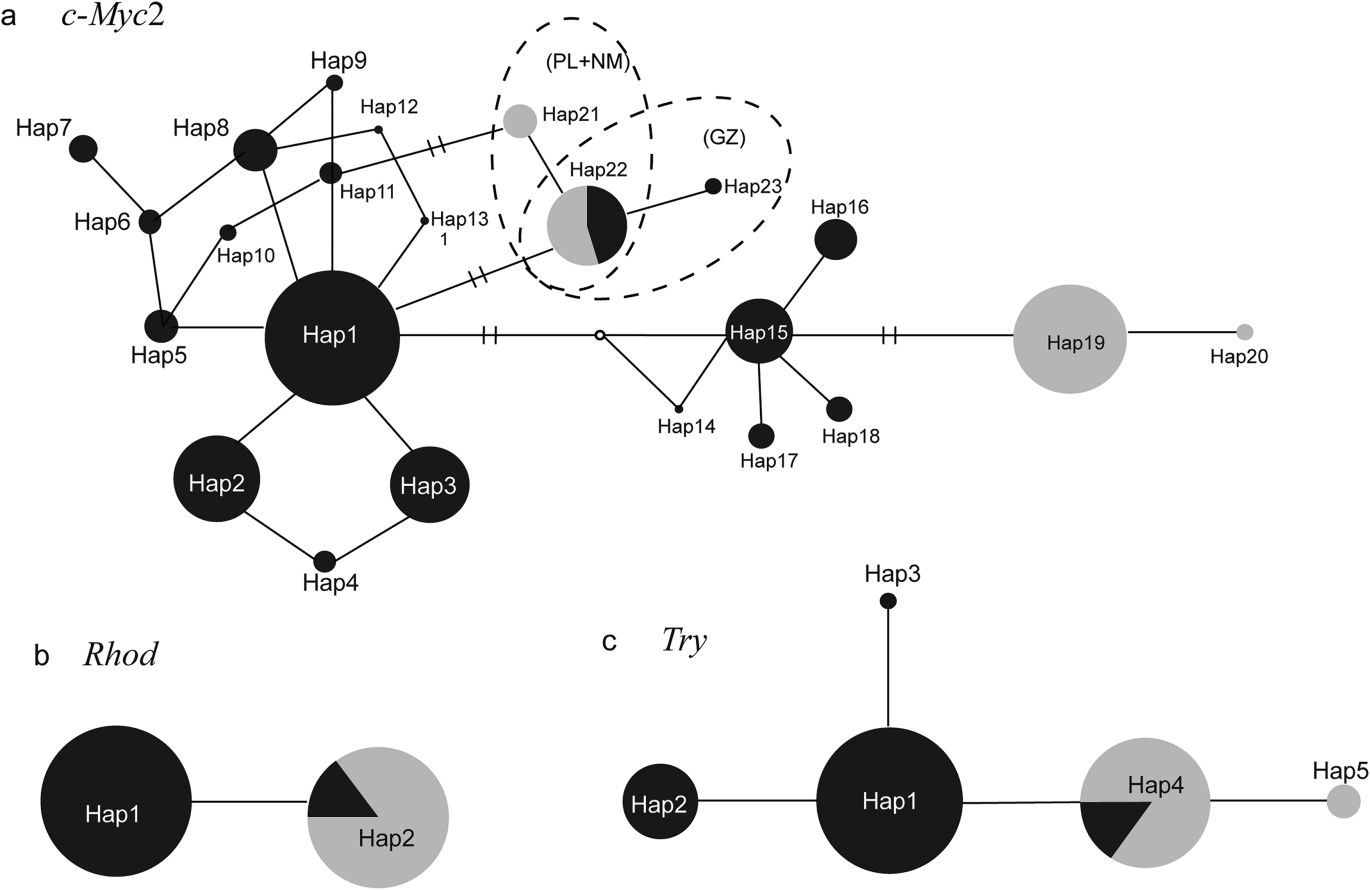
Median-joining networks for haplotype relationships of (a) *c-Myc2,* (b) *Rhod* and (c) *Tyr* in *N. parkeri.* The haplotypes and their frequencies are labeled. The black and grey colours represent the populations from eastern and western regions, respectively (Table 1). Network branches linking the cycles indicate one mutation step; two or more mutations are represented by slashes crossed with the network branches. The small open circle represents hypothetical missing intermediate.

Although the three of the nuclear genes did not reveal well-supported haplogroups, the eastern and western populations had their own distinct haplotypes in each gene with few exceptions (Fig. 3). For *c-Myc*2, four haplotypes (Hap19–22) were determined in the eight western populations, and Hap21 and Hap22 derived from populations NM and PL were very different from Hap19 and Hap20 from the rest western populations (Fig. 3a). Furthermore, all the individuals in PL had Hap22, a haplotype common to the individuals from the eastern population GZ (Fig. 3a). All the *Rhod* sequences collapsed into two haplotypes: Hap1 was derived from eastern populations, while all the western-region populations had Hap2 (Fig. 3b). All but one individual from GZ and several individuals from YB in the eastern region also had Hap2. Similarly, of the five haplotypes detected in *Tyr*, Hap1-Hap3 belonged to eastern populations, while Hap4 and Hap5 belonged to western populations (Fig. 3c). All but one individual from GZ shared Hap4 with all the western populations.

### Divergence time

Divergence time for the Cytb key nodes was calculated with a slower^32^ (6.9 × 10^-9^/site/year) and a much faster^33^ (1.8 × 10^-8^/site/year) mutation rate, respectively. With the slower mutation rate, divergence time for the deep split between eastern and western populations was dated back to 3.72 million years ago (Mya) with 95% highest posterior density (95% HPD) of 2.72–4.74 Mya (Fig. 2). Divergence within lineages East and West were traced back to 0.94 (95% HPD: 0.59–1.31) and 1.71 (95% HPD: 1.08–2.38) Mya, respectively. With the higher mutation rate, the deep split was dated to 1.43 (95% HPD: 1.04–1.83) Mya. Lineages East and West split about 0.36 (95% HPD: 0.23–0.50) and 0.66 (95% HPD: 0.42–0.91) Mya, respectively.

### Demographic history

Tajima’s *D* values in neutrality tests were negative and significant (*P* < 0.05) in lineages East and West, as well as in sublineages E1 and W1 (Table 2), suggesting past population expansion. Significant (*P* < 0.01) Fu’s *F_S_*were detected in lineage East, sublineages E1, E2 and W1. The demographic trend for the sublineage W2 was not investigated in the mismatch and BSP analyses because of the small sample size (*n* = 9). In the analysis of mismatch distribution, SSD and H_rag_ values supported the population expansion model in all the Cyt*b* lineages and sublineages (excluding lineage East). Sublineages E1– E4 and W1 each exhibited an observed unimodal mismatch frequency distribution (Supplementary Fig. S2 online), fitting a sudden population expansion model. The BSP analyses revealed a past increase of effective population size in all the lineages and sublineages.

### Species distribution modelling

**Figure 4:**
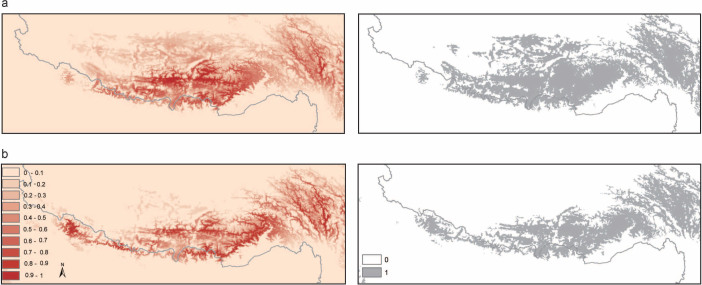
Predicted species distributions of *N. parkeri* under (a) current conditions, and (b) LGM conditions estimated from the CCSM climate models. The right column indicates the suitable (1) and unsuitable (0) habitats by thresholds of the mean maximum training sensitivity plus specificity. The maps were produced using ArcGIS v10.1 (ESRI, CA, USA) and Adobe Illustrator CS5 v15.0.0 (Adobe Systems Inc., San Francisco, CA).

We used species distribution models (SDMs) to predict the potential habitat distribution of *N. parkeri* under present and the Last Glacial Maximum (LGM) conditions. The Maximum Entropy (Maxent) model worked well in predicting occurrences under current conditions. The average AUC (area under the receiver operating characteristic curve) value for the test data across 4 runs was 0.92 with a standard deviation of 0.02. Averaged distributions under present and the LGM conditions are shown in Fig. 4. The SDM under present conditions corresponded well to the current known distribution of *N. parkeri* on the Tibetan Plateau (Fig. 4a). During the LGM, the predicted distribution showed a slight contraction in northwestern and northern regions of the plateau platform compared with the current distribution (Fig. 4b).

## Discussion

In this study, two major Cyt*b* lineages (East and West) were resolved in *N. parkeri* populations. Generally there were no genetic and geographic overlaps between these two lineages. The maternal split was confirmed by three nuclear genes in that eastern and western populations had distinct nuclear haplotypes. The estimated split time indicated an old divergence (1.4–3.7 Mya) between the two lineages. Therefore, this deep divergence suggests a long history of population isolation, wherein each group could represent a distinct lineage.

Considering the heterogeneous landscape in the southern region of the Tibetan Plateau and the weak dispersal capacity of the species studied, geographic barriers (e.g. high mountains or deep rivers) might have contributed to this deep divergence. However, there are no apparent geographic barriers between the two regions. Furthermore, widespread distribution of the Cyt*b* E1 and W1 lineages (Fig. 1) suggested that topography alone cannot produce and maintain the deep divergence. Alternatively, Pleistocene glaciations, which accounted for long-term population isolation and divergence in many modern species^34^, could contribute to the observation here. Up to four Pleistocene glaciations have been identified in the Tibetan Plateau^11^,^35^,^36^. The oldest glaciation reportedly occurred at 1.17–0.8 Mya^11^, but was limited to several marginal mountains^36^. There is no evidence that the frog populations were isolated in the marginal regions and dispersed to the plateau platform (see below). The maximum glaciation occurred during *ca*. 0.72–0.5 Mya, and the following glaciations became less geographically extensive^11^,^36^, which were long after the deep divergence. Thus, the Pleistocene glaciations on the Tibetan Plateau seem not to account for the oldest genetic split. Interestingly, the boundary between the two lineages was roughly along the 400 mm annual precipitation^37^. Thus, the genetic split might be ascribed to climatic and ecological factors. The plateau have been arid and dry since the uplift of the Tibetan Plateau and the subsequent Pleistocene glaciations^36^. The eastern region of the southern Tibetan Plateau that the populations of the Lineage East occupy is dominated by the southwest monsoon, making it the most moist area in the plateau^37^, whereas areas to the west and north are much more arid and drier. The shifting of the climatic zones might be barriers to the migration and dispersal for this frog. Similar genetic divergence patterns have been observed in shrubs in this region^17^, which was also thought to be shaped by the climatic and ecological factors.

It was argued that populations surviving in refugia always show higher genetic diversity than the post-glacial colonized/recolonized populations because of long-term persistence and population structure^1^,^2^. In other words, genetic diversity is expected to decrease along the colonization/recolonization routes^38^. If there were southeastern or eastern refugia at the edge of the Tibetan Plateau, the genetic diversity would decrease from the refugial populations to those recently colonized/recolonized in the western and northern interior parts. However, this is not the case in our study. Populations exhibiting high or moderate Cyt*b* haplotype diversity (Supplementary Table S2), e.g. NM, CN, NY, SP, were all located along the Yarlung Zangbo River and Kyichu River (i.e. Lhasa River). There was no evidence of genetic diversity decline from the plateau edge to the interior platform. Furthermore, the fact that a large proportion (92%) of private Cyt*b* haplotypes was identified in 24 of all the 30 populations strongly rejected the hypothesis of low-altitude peripheral refugia.

Instead, several lines of evidence support the scenario of multiple glacial refugia on the plateau platform. First, two deep genetic lineages were revealed, dating back to the Middle Pleistocene or earlier, long before the LGM. Although the divergence time should be interpreted with caution in absence of fossil records, these genetic lineages probably survived the maximum glaciation^11^,^36^ (*c.* 0.72–0.5 Mya) and several following less extensive glaciations on the plateau. If they survived in one glacial refugia, then the genetic lineages would have mixed together in terms of nuclear genes. On the contrary, the two lineages had distinct nuclear haplotypes, implying that they survived in different refugia during the glacial periods. Second, refugia always provided suitable stable habitats for extant organisms through geological time scales (Graham *et al.*, 2010). Together with the current occurrence, the SDMs indicated that the Yarlung Zangbo valley and Kyichu River catchment might be the most suitable habitats for *N. parkeri* (Figs. 4 and S1) during LGM and current climatic conditions. Recent studies have proved that these regions served as refugia for plants (Opgenoorth *et al.*, 2010; Wang *et al.*, 2010) and animals (Hofmann, 2012) during glaciations. Third, this scenario fits well with the Pleistocene glacier conditions on the Tibetan Plateau where extensive ice caps and valley glaciers formed during the glaciations (Shi, 2002; Owen *et al.*, 2008). A recent study with sedimentological, geochronological and palaeobotanical analyses revealed that the Kyichu River was free of ice in the Last Glacial (~0.034 Mya), before LGM^39^. Such ice-free regions could provide refugia for many species to survive.

Two distinct genetic lineages were detected, but several exceptions were also observed from the populations in the boundary region (Figs. 1 and 3). In this study, postglacial expansion was detected for *N. parkeri*. In particular, the Cyt*b* sublineages E1 and W1 showed very strong signals of postglacial expansion in the neutrality tests, mismatch distribution and BSP analyses. It is, therefore, plausible to postulate that the boundary region was probably a second contact zone. A finer analysis based on more specimens should be performed to better understand the microevolutionary processes in this boundary region.

The apparent genetic structure and limited gene flow between the eastern and western populations has important conservation implications for *N. parkeri*. These two distinct genetic lineages, which diverged for a long time and had limited gene flow between them, could be considered as two evolutionarily significant units (ESUs), and each needs to be protected. The Yarlung Zangbo valley and the Kyichu catchment were thought to be the potential refugia for this frog. These regions, in particular the Lhasa area (within the Kyichu River catchment), have been strongly affected by human activity as early as four thousand years ago^39^. These potential refugia habitats require more effort and management for the protection.

In summary, our data provide comprehensive genetic evidence of anurans on the interior Tibetan Plateau that paleoclimatic events during the Quaternary have a great influence on the distribution and evolution of alpine organisms. Results of phylogeographic structure and ecological modeling analyses supported the hypothesis of multiple refugia on the plateau platform, with potential refugia in the Yarlung Zangbo valley and Kyichu catchment. Our study also provides useful genetic information essential for the conservation of this endemic frog.

## Methods

The methods were carried out in accordance with the approved guidelines of the Good Experimental Practices adopted by the Institute of Zoology, Chinese Academy of Sciences. All experimental procedures and animal collection were conducted under the permits (No. IOZ13015) approved by the Committee for Animal Experiments of the Institute of Zoology, Chinese Academy of Sciences, China.

### Sample collection, DNA extraction and sequencing

*N. parkeri* adults were collected by hand from shallow ponds on the Tibetan Plateau during the breeding season (July-August) in 2012. Tissue samples (toe clips from back legs) were taken and stored in 95–100% ethanol. All frogs were released back into the wild at the capture sites immediately after sampling without harming. Total genomic DNA was extracted from the tissues using a standard phenol-chloroform extraction procedure after digestion with proteinase K^40^.

One mitochondrial (cytochrome *b*, Cyt*b*) and three nuclear fragments (cellular myelocytomatosis intron 2, *c-Myc2*; rhodopsin, *Rhod*; tyrosinase, *Tyr*) were sequenced for molecular analyses. The partial Cyt*b* sequences were amplified with primers Cytb-F and Cytb-R, and the fragments of *c-Myc2* with CYMC5 and CYMC6^41^, *Rhod* with Rhod1A and Rhod1D, and *Tyr* with Tyr1G and Tyr1B^42^. Summary information of the primer sets and their annealing temperatures (T_m_) in polymerase chain reactions (PCRs) are detailed in Supplementary Table S4 online. PCRs were performed in a 50 µL reaction volume containing 25 µL Premix Ex Taq II Version 2.0 [2×; Takara Biotechnology (Dalian) Co., Ltd], 0.3 µM of each PCR primer, and 80 ng of genomic DNA. The PCR protocols were: an initial denaturation at 94°C for 5 min, followed by 28 cycles at 94°C for 40 s, Tm (see Table S4 online) for 40 s, 72°C for 75 s, and a final extension at 72°C for 10 min. The PCR products were sequenced on an ABI 3730 capillary sequencer (Applied Biosystems, Life technologies, NY, USA) using a BigDye Terminator v3.1 Cycle Sequencing Kit (Life technologies, NY, USA). The three nuclear genes were sequenced directly in the forward direction, and mitochondrial Cyt*b* fragments were sequenced in both directions.

Mitochondrial sequences were viewed and edited with the program DNAStar SeqMan v7.21 (DNAStar Inc., Madison, WI, USA). All sequences were corrected manually, aligned and trimmed to uniform lengths using BioEdit v7.1.8^43^ under default settings. The program DAMBE v5.3.15^44^ was used to determine the haplotypes. For nuclear sequences, recombination was determined using the seven methods (RDP, GEnECONV, Bootscan, MaxChi, Chimaera, SiScn and 3Seq) implemented in RDP4^45^. No recombination was observed in the nuclear sequences. A Bayesian framework in PHASE v2.1^46^ was applied to infer the haplotypic state of nuclear genes with a probability threshold of 90% and five independent runs. This computational method has been proved to be accurate in haplotype reconstruction^47^,^48^. The input files for PHASE were prepared by the web tool SeqPHASE^49^ (http://seqphase.mpg.de/seqphase/).

### Phylogenetic analyses

Phylogenetic topologies of Cyt*b* haplotypes were reconstructed using Bayesian inference (BI) and maximum likelihood (ML) methods. A homologous fragment from the complete mitochondrial genome of *N. pleskei* (NC_016119), a closely related species to *N. parkeri*, was used as the outgroup. The Cyt*b* sequences were partitioned by codon position. The best-fit model of nucleotide substitutions for each partition was estimated from 88 models using the ML topology optimization approach under the Bayesian information criteria (BIC) as implemented in jModelTest v0.1.1^50^. For the first, second and third codon regions, the best models were K80+I, F81 and TN93+G, respectively. Partitioned Bayesian analyses were carried out using MrBayes v3.1.2^51^. Variable rates across data partitions were used. Two independent runs with four (one cold and three incrementally heated) Markov chain Monte Carlo (MCMC) chains were performed with a temperature of 0.25. Chains were run for 10,000,000 generations, sampling every 1,000 generations. Convergence was assessed by measuring average standard deviations of split frequencies (< 0.01). The effective sample size (ESS) for each parameter (> 200) was estimated by Tracer v1.6 (http://tree.bio.ed.ac.uk/software/tracer/) to assess the stationarity and convergence. The first 25% of the sampled trees were discarded as burn-in, and the remaining trees were used to construct a majority rule consensus tree and assess the posterior probabilities. The default values were used for the other parameters. The ML method based on the partitioned data was performed in RaxML v7.2.6^52^ via the graphical interface raxmlGUI v1.3^53^. The GTR+G+I model was applied to the three partitions. Node support values were estimated by the rapid bootstrap with 1,000 pseudoreplicates.

For nuclear genes, because of the limited number of the parsimony informative sites, phylogenetic topologies were only constructed in *c-Myc2* by using the BI method. JC+G model was chosen as the best-fitting model under BIC by jModelTest. MCMC chains were run for 5,000,000 generations, and the rest parameters were set as default.

### Population genetic analyses

Nucleotide (π) and haplotype diversity (*h*) for each Cyt*b* lineages (see Results) and population were calculated using DNAsp v5.10.00^54^. Genetic distances (uncorrected *p*-distance) between Cyt*b* lineages were computed in Mega v5.1^55^. Population structure was assessed by a hierarchical analysis of molecular variance (AMOVA) using Φ-statistics. Populations within the eastern and western regions (Table 1) were pooled into two groups. Pairwise Φ_ST_ was estimated between Cyt*b* sublineages. Statistical significance was tested by 10,000 non-parametric permutations at the 5% significance level. Both analyses were implemented in Arlequin v3.5^56^. To visualize relationships among haplotypes, median-join networks^57^ were constructed with Network v4.6.1.1 (http://www.fluxus-engineering.com/) for the mitochondrial (Cyt*b*) and nuclear (*c-Myc2,* *Rhod* and *Tyr*) haploytpes, respectively.

### Divergence time estimation

The time to the most recent common ancestor (TMRCA) for the key nodes in Cyt*b* was estimated using a Bayesian MCMC approach in BEAST v1.7.5^58^. A likelihood ratio test (LRT) was employed to test whether th*e* Cyt*b* data evolved under a molecular clock or not. Likelihood scores were calculated with and without enforcing a molecular clock model in the program PAUP * v4.0b10^59^, and significance of the likelihood ratio test was determined by a chi-square test. The global molecular clock model was not rejected (χ^2^ = 41.4, d.f. = 73, *P* > 0.05). An evolutionary rate of 6.9 × 10^-9^/site/year in mitochondrial genes has been estimated in bufonid species^32^, and similar estimates of molecular clock have been used in other anuran species^60^,^61^,^62^,^63^,^64^. Also, a much faster mutation rate of 1.8 × 10^-8^/site/year was used in Cyt*b* of Ranid species^33^. To get a conservative framework of the divergence time in *N. parkeri*, we conducted the analyses by using both the mutation rates, respectively. The strict molecular clock with the HKY+G substitution model selected by jModelTest was used and the constant population size coalescent was set as the tree prior. The MCMC chains were run for 30,000,000 steps and the parameters were logged every 3,000 steps. Three independent runs were performed. The results were combined with LogCombiner v1.7.5 from the BEAST package, and then visualized in Tracer v1.6 with burning in 10% of the original chain length.

### Demographic history

Demographic histories were assessed in three different ways. We first calculated Tajima’s *D*^65^ and Fu’s *F_S_*^66^ to assess population expansion. These neutrality tests were employed in DnaSP. Significantly (*P* < 0.05) negative values for these statistics indicate past population expansions. Secondly, a pairwise mismatch distribution analysis, which considers the distribution of pairwise differences between haplotypes, was conducted in Arlequin with 10,000 permutations. For each Cyt*b* lineage, theoretical distribution under the sudden expansion model was compared to the observed data. Goodness-of-fit was tested between the observed and the expected distribution of pairwise differences for each lineage using the sum of squared deviation (SSD) and Harpending’s raggedness index (H_rag_)^67^. Finally, a Bayesian skyline plot (BSP) analysis was implemented in BEAST to estimate the change in population size over time. A substitution rate of Cyt*b* ranging from 6.9 × 10^-9^/site/year^32^ to 1.8 × 10^-8^/site/year^33^ was applied. The parameters were set as described above except that Bayesian skyline was used for the tree prior.

### Species distribution modelling

The species distribution models (SDMs) were generated using the maximum entropy machine-learning algorithm as implemented in the program Maxent v3.3.3k^68^. The occurrence data of *N. parkeri* from the GBIF database (Global Biodiversity Information Facility, http://data.gbif.org), literatures^69^,^70^,^71^ and the sampling locations were collected, and a total of 34 points (Supplementary Table S5) were used in the final analyses.

A set of 19 bioclimatic variables for both current (~1950–2000) and the Last Glacial Maximum (LGM, ~21000 years before present) conditions were downloaded from the WorldClim v1.4 database (http//www.worldclim.org) with a resolution of 2.5 arc-minutes. These variables describe temperature and precipitation^72^. Layers of the LGM conditions were obtained by the community climate system model (CCSM). To minimize model over-fitting, pairwise correlations between variables were assessed with Pearson’s correlation coefficient (*r*). When two variables were strongly correlated (*r* > 0.9), we chose the one being more biologically meaningful for the species^73^. In total, nine bioclimatic variables, i.e. annual mean temperature, mean diurnal range, Isothermality, temperature seasonality, temperature annual range, annual precipitation, precipitation of driest month, precipitation seasonality, and precipitation of driest quarter, were used in the subsequent analyses.

SDMs were constructed using fourfold cross-validation, with 25% of occurrence points as test data. The maximum iteration was set to 5000 and the other parameters were set to the default values. Model performance was assessed through comparison of the area under the receiver operating characteristic curve (AUC) values for training and test data. An AUC of 0.5 indicates a random prediction of presence and absence, while an AUC of 1 represents a perfect discrimination. The mean maximum training sensitivity plus specificity was used as the threshold to discriminate suitable from unsuitable habitats, which has been proved recently an accurate approach^74^. All geographical information system (GIS) operations were performed in the software ArcGIS v10.1 (ESRI, CA, USA).

## Author Contributions

M.-H.L. and J.L. designed the study. J.L. analysed and interpreted the data, and wrote the manuscript. X.H., X.X., P.L. and Q.Z. participated in the sample collection. C.W. and D.F. conducted the laboratory work. M.-H.L. and J.L. revised the manuscript. All the authors read and approved this paper.

## Additional Information

**How to cite this article**: Liu, J. *et al*.Phylogeography of Nanorana parkeri (Anura: Ranidae) and multiple refugia on the Tibetan Plateau revealed by mitochondrial and nuclear DNA. *Sci. Rep.* **5**, 9857; doi: 10.1038/srep09857 (2015).

## Supplementary Material

Supplementary Information Supplementary Information

## Figures and Tables

**Table 1:** Sampling populations with the geological information and the number of sequences and haplotypes for the Cyt*b*, *c-Myc*2, *Rhod* and *Tyr* fragments in each population.

**Table 2:** Genetic diversity, neutrality tests and mismatch goodness-of-fit tests in the Cyt*b* lineages. Number of sequences and haplotypes in each lineage are shown as well. The demographic trend for the sublineage W2 was not investigated in the mismatch analyses because of the small sample size (*n* = 9).

**Table 3:** Results of analysis of molecular variance (AMOVA) of Cyt*b* data. Regions refer to the eastern and western regions (Table 1).

Source of variation	*d.f.*	Sum of squares	Variance component	Variation (%)	Fixation indices
Between regions	1	3799.446	20.22861	93.22	Φ_CT_: 0.93**
Among populations within regions	28	470.345	0.92118	4.25	Φ_SC: _0.63**
Within populations	502	276.256	0.55031	2.54	Φ_ST_: 0.97**
Total	531	4546.047	21.70009		

*d.f.*, degrees of freedom.

**denotes significance at α = 0.01.
