# Supplementary material for: Phylogeography of Nanorana parkeri (Anura: Ranidae) and multiple refugia on the Tibetan Plateau revealed by mitochondrial and nuclear DNA
Source: Sci Rep. 2015 May 18;5:9857. doi: 10.1038/srep09857 (PMC4434895; doi:10.1038/srep09857)
Supplement: Supplementary Information [file srep09857-s1.pdf]

Phylogeography of *Nanorana parkeri* (Anura: Ranidae) and multiple refugia on the Tibetan Plateau revealed by mitochondrial and nuclear DNA

Supplementary Information

Authors: Jun Liu[1,2], Cuimin Wang[1,3], Dongli Fu[4], Xiaoju Hu[1,3], Xiangmo Xie[1,5], Pengfei Liu[1,3], Qiong Zhang[1] and Meng-Hua Li[1*]

[1] CAS Key Laboratory of Animal Ecology and Conservation Biology, Institute of Zoology, Chinese Academy of Sciences (CAS), Beijing 100101, China

[2] Deep-Sea Research Department, Sanya Institute of Deep-Sea Science and Engineering, Chinese Academy of Sciences (CAS), Sanya 572000, China

[3] College of Life Sciences, University of the Academy of Sciences, Beijing 100049, China

[4] Gansu Key Laboratory of Herbivorous Animal Biotechnology, Gansu Agricultural University, Lanzhou 730070, Chinas

[5] College of Life Sciences, Yangtze University, Jingzhou 434025, Hubei, China

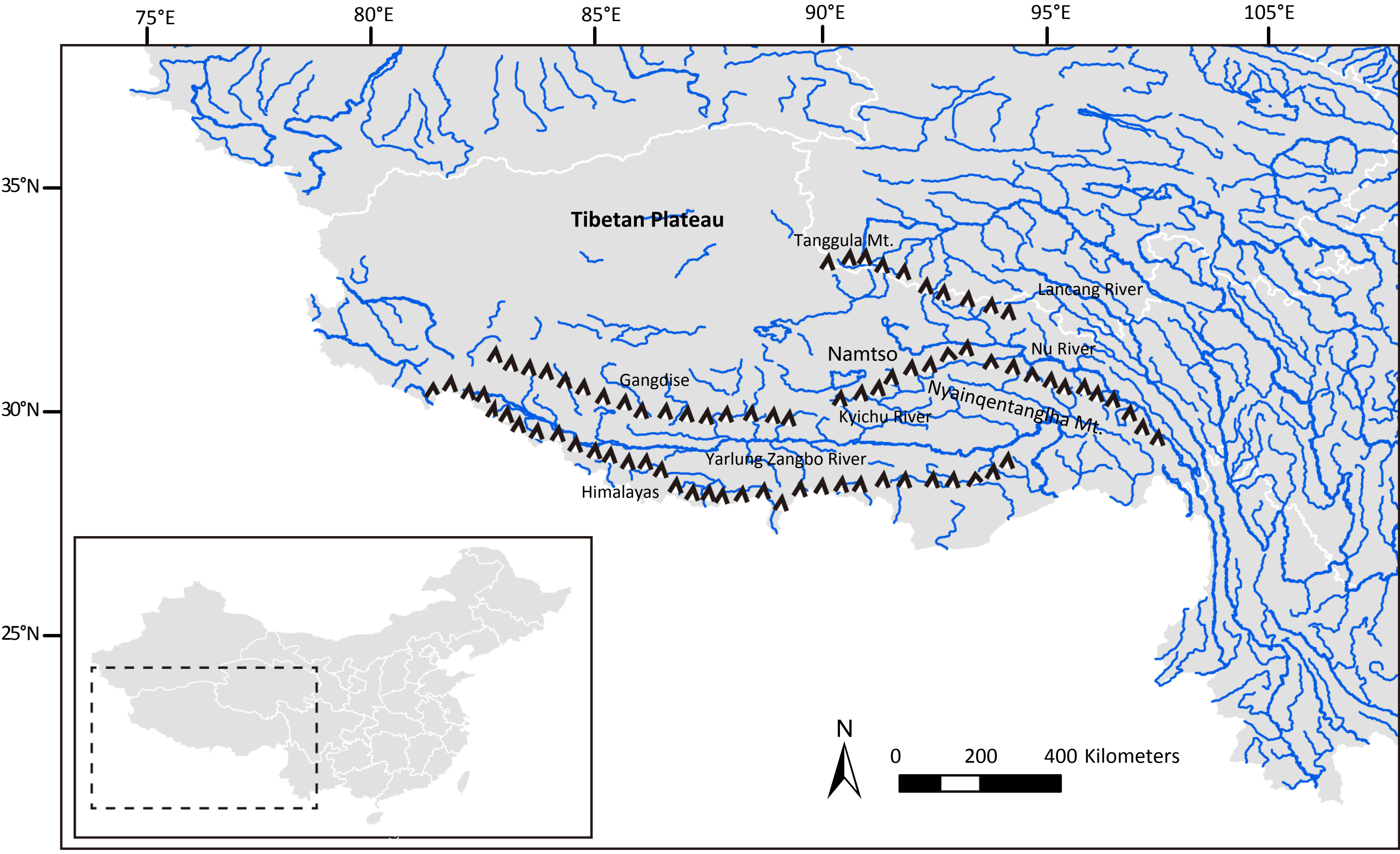

Figure S1 Simplified map exhibiting mountains and rivers in the southern Tibetan Plateau. The map was generated using ArcGIS v10.1 (ESRI, CA, USA) and Adobe Illustrator CS5 v15.0.0 (Adobe Systmes Inc., San Francisco, CA).

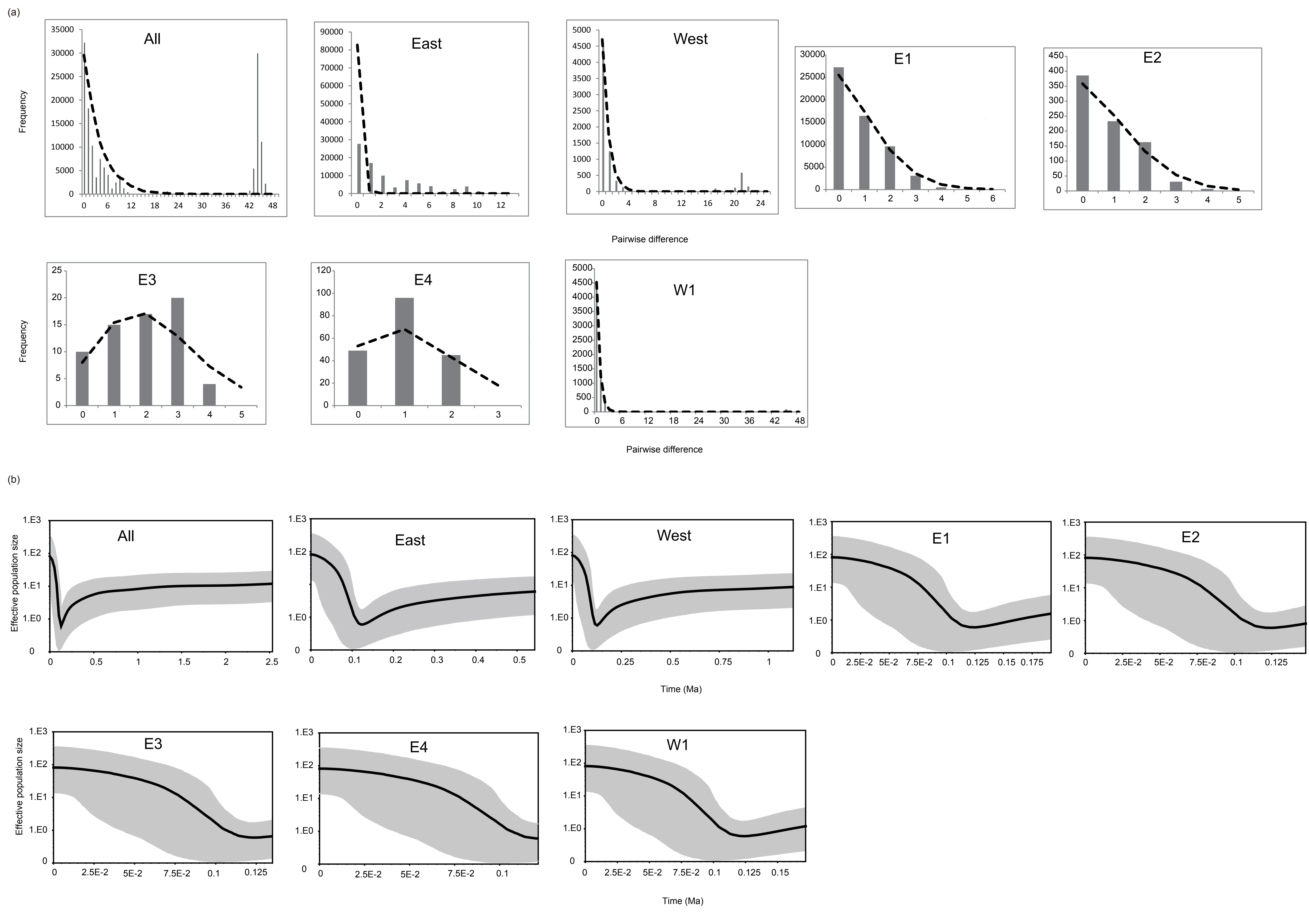

(a)
All
East
West
E1
E2
E3
E4
W1
Frequency
Pairwise difference
(b)
All
East
West
E1
E2
E3
E4
W1
Effective population size
Time (Ma)

Figure S2 Mismatch distributions (a) and Bayesian skyline plot (BSP) analyses (b) for the Cyt*b* (sub)lineages. The demographic trend for the sublineage W2 was not investigated in the mismatch and BSP analyses because of the small sample size (n = 9). Bars in the mismatch distributions represent the observed frequency of pairwise differences, and the dashed lines indicate the expected distribution under a model of population expansion. In the BSP, the thick solid line depicts the median estimate and the shading is the 95% highest posterior density (HPD) limits. The Y-axis (logarithmic scale) represents effective population size. The X-axis is the time-scale in million years ago (Ma).

Table S1 Detailed information of GenBank accession numbers and haplotypes (Hap) of Cyt*b*, *c-Myc2*, *Rhod* and *Tyr* for all the samples used in this study. For the abbreviation of each population see Table 1.

	Sample	Cyt*b*		*c-Myc2*		*Rhod*		*Tyr*	
Population	ID	Hap	GenBank	Hap	GenBank	Hap	GenBank	Hap	GenBank
RW	RW-1	Hap42	KJ434229	Hap1	KJ434263	Hap1	KJ434286	Hap1	KJ434288
	RW-2	Hap42	KJ434229	Hap1	KJ434263	Hap1	KJ434286	Hap1	KJ434288
	RW-3	Hap42	KJ434229	Hap1	KJ434263	Hap1	KJ434286	Hap1	KJ434288
	RW-4	Hap37	KJ434224	Hap1	KJ434263	Hap1	KJ434286	Hap1	KJ434288
	RW-5	Hap42	KJ434229	Hap1	KJ434263	Hap1	KJ434286	Hap1	KJ434288
	RW-6	Hap42	KJ434229	Hap1	KJ434263	Hap1	KJ434286	Hap1	KJ434288
	RW-7	Hap42	KJ434229	Hap1	KJ434263	Hap1	KJ434286	Hap1	KJ434288
	RW-8	Hap42	KJ434229	Hap1	KJ434263	Hap1	KJ434286	Hap1	KJ434288
	RW-9	Hap42	KJ434229	Hap1	KJ434263	Hap1	KJ434286	Hap1	KJ434288
	RW-10	Hap42	KJ434229	Hap1	KJ434263	Hap1	KJ434286	Hap1	KJ434288
	RW-11	Hap37	KJ434224	Hap1	KJ434263	Hap1	KJ434286	Hap1	KJ434288
	RW-12	Hap42	KJ434229	Hap1	KJ434263	Hap1	KJ434286	Hap1	KJ434288
	RW-13	Hap42	KJ434229	Hap1	KJ434263	Hap1	KJ434286	Hap1	KJ434288
	RW-14	Hap42	KJ434229	Hap1	KJ434263	Hap1	KJ434286	Hap1	KJ434288
	RW-15	Hap37	KJ434224	Hap1	KJ434263	Hap1	KJ434286	Hap1	KJ434288
	RW-16	Hap42	KJ434229	Hap1	KJ434263	Hap1	KJ434286	Hap1	KJ434288
	RW-17	Hap37	KJ434224	Hap1	KJ434263	Hap1	KJ434286	Hap1	KJ434288
	RW-18	Hap42	KJ434229	Hap1	KJ434263	Hap1	KJ434286	Hap1	KJ434288
	RW-19	Hap42	KJ434229	Hap1	KJ434263	Hap1	KJ434286	Hap1	KJ434288
	RW-20	Hap42	KJ434229	Hap1	KJ434263	Hap1	KJ434286	Hap1	KJ434288
	RW-21	Hap42	KJ434229	Hap1	KJ434263	Hap1	KJ434286	Hap1	KJ434288
	RW-22	Hap42	KJ434229	Hap1	KJ434263	Hap1	KJ434286	Hap1	KJ434288
	RW-23	Hap42	KJ434229	Hap1	KJ434263	Hap1	KJ434286	Hap1	KJ434288
	RW-24	Hap42	KJ434229	Hap1	KJ434263	Hap1	KJ434286	Hap1	KJ434288
JJ	JJ-1	Hap42	KJ434229	Hap1	KJ434263	Hap1	KJ434286	Hap1	KJ434288
	JJ-2	hap11	KJ434198	Hap1	KJ434263	Hap1	KJ434286	Hap1	KJ434288
	JJ-3	Hap42	KJ434229	Hap1	KJ434263	Hap1	KJ434286	Hap1	KJ434288
	JJ-4	Hap42	KJ434229	Hap1	KJ434263	Hap1	KJ434286	Hap1	KJ434288
				Hap8	KJ434270				
	JJ-5	Hap27	KJ434214	Hap1	KJ434263	Hap1	KJ434286	Hap1	KJ434288
	JJ-6	Hap42	KJ434229	Hap1	KJ434263	Hap1	KJ434286	Hap1	KJ434288
	JJ-7	hap12	KJ434199	Hap1	KJ434263	Hap1	KJ434286	Hap1	KJ434288
	JJ-8	Hap28	KJ434215	Hap1	KJ434263	Hap1	KJ434286	Hap1	KJ434288
	JJ-9	Hap42	KJ434229	Hap1	KJ434263	Hap1	KJ434286	Hap1	KJ434288
	JJ-10	Hap42	KJ434229	Hap1	KJ434263	Hap1	KJ434286	Hap1	KJ434288
	JJ-11	Hap42	KJ434229	Hap1	KJ434263	Hap1	KJ434286	Hap1	KJ434288

	JJ-12	Hap42	KJ434229	Hap1	KJ434263	Hap1	KJ434286	Hap1	KJ434288
	JJ-13	Hap36	KJ434223	Hap1	KJ434263	Hap1	KJ434286	Hap1	KJ434288
	JJ-14	Hap42	KJ434229	Hap1	KJ434263	Hap1	KJ434286	Hap1	KJ434288
	JJ-15	Hap42	KJ434229	Hap1	KJ434263	Hap1	KJ434286	Hap1	KJ434288
	JJ-16	Hap42	KJ434229	Hap1	KJ434263	Hap1	KJ434286	Hap1	KJ434288
	JJ-17	Hap42	KJ434229	Hap1	KJ434263	Hap1	KJ434286	Hap1	KJ434288
	JJ-18	Hap26	KJ434213	-	-	Hap1	KJ434286	Hap1	KJ434288
	JJ-19	Hap29	KJ434216	Hap1	KJ434263	Hap1	KJ434286	Hap1	KJ434288
	JJ-20	Hap42	KJ434229	Hap1	KJ434263	Hap1	KJ434286	Hap1	KJ434288
	JJ-21	Hap42	KJ434229	Hap2	KJ434264	Hap1	KJ434286	Hap1	KJ434288
	JJ-22	Hap42	KJ434229	-	-	Hap1	KJ434286	Hap1	KJ434288
ZB	ZB-1	Hap68	KJ434255	Hap19	KJ434281	Hap2	KJ434287	Hap4	KJ434291
	ZB-2	Hap68	KJ434255	Hap19	KJ434281	Hap2	KJ434287	Hap4	KJ434291
	ZB-3	Hap68	KJ434255	Hap19	KJ434281	Hap2	KJ434287	Hap4	KJ434291
	ZB-4	Hap68	KJ434255	Hap19	KJ434281	Hap2	KJ434287	Hap4	KJ434291
	ZB-5	Hap68	KJ434255	Hap19	KJ434281	Hap2	KJ434287	Hap4	KJ434291
	ZB-6	Hap68	KJ434255	Hap19	KJ434281	Hap2	KJ434287	Hap4	KJ434291
	ZB-7	Hap68	KJ434255	Hap19	KJ434281	Hap2	KJ434287	Hap4	KJ434291
	ZB-8	Hap68	KJ434255	Hap19	KJ434281	Hap2	KJ434287	Hap4	KJ434291
	ZB-9	Hap64	KJ434251	Hap19	KJ434281	Hap2	KJ434287	Hap4	KJ434291
	ZB-10	Hap68	KJ434255	Hap19	KJ434281	Hap2	KJ434287	Hap4	KJ434291
	ZB-11	Hap68	KJ434255	Hap19	KJ434281	Hap2	KJ434287	Hap4	KJ434291
	ZB-12	Hap68	KJ434255	Hap19	KJ434281	Hap2	KJ434287	Hap4	KJ434291
	ZB-13	Hap68	KJ434255	Hap19	KJ434281	Hap2	KJ434287	Hap4	KJ434291
	ZB-14	Hap68	KJ434255	Hap19	KJ434281	Hap2	KJ434287	Hap4	KJ434291
	ZB-15	Hap68	KJ434255	Hap19	KJ434281	Hap2	KJ434287	Hap4	KJ434291
	ZB-16	Hap68	KJ434255	-	-	Hap2	KJ434287	Hap4	KJ434291
	ZB-17	Hap68	KJ434255	Hap19	KJ434281	Hap2	KJ434287	Hap4	KJ434291
	ZB-18	Hap68	KJ434255	Hap19	KJ434281	Hap2	KJ434287	Hap4	KJ434291
	ZB-19	Hap68	KJ434255	Hap19	KJ434281	Hap2	KJ434287	Hap4	KJ434291
	ZB-20	Hap68	KJ434255	Hap19	KJ434281	Hap2	KJ434287	Hap4	KJ434291
DQ	DQ-1	Hap15	KJ434202	Hap1	KJ434263	Hap1	KJ434286	Hap1	KJ434288
	DQ-2	Hap15	KJ434202	Hap1	KJ434263	Hap1	KJ434286	Hap1	KJ434288
	DQ-3	Hap15	KJ434202	Hap1	KJ434263	Hap1	KJ434286	Hap1	KJ434288
	DQ-4	Hap42	KJ434229	Hap1	KJ434263	Hap1	KJ434286	Hap1	KJ434288
	DQ-5	Hap42	KJ434229	Hap1	KJ434263	Hap1	KJ434286	Hap1	KJ434288
	DQ-6	Hap42	KJ434229	Hap1	KJ434263	Hap1	KJ434286	Hap1	KJ434288
	DQ-7	Hap15	KJ434202	Hap1	KJ434263	Hap1	KJ434286	Hap1	KJ434288
	DQ-8	Hap15	KJ434202	Hap1	KJ434263	Hap1	KJ434286	Hap1	KJ434288
	DQ-9	Hap42	KJ434229	Hap1	KJ434263	Hap1	KJ434286	Hap1	KJ434288
	DQ-10	Hap15	KJ434202	Hap1	KJ434263	Hap1	KJ434286	Hap1	KJ434288
	DQ-11	Hap15	KJ434202	Hap1	KJ434263	Hap1	KJ434286	Hap1	KJ434288

	DQ-12	Hap42	KJ434229	Hap1	KJ434263	Hap1	KJ434286	Hap1	KJ434288
	DQ-13	Hap15	KJ434202	Hap1	KJ434263	Hap1	KJ434286	Hap1	KJ434288
	DQ-14	Hap15	KJ434202	Hap1	KJ434263	Hap1	KJ434286	Hap1	KJ434288
	DQ-15	Hap16	KJ434203	Hap1	KJ434263	Hap1	KJ434286	Hap1	KJ434288
	DQ-16	Hap15	KJ434202	Hap1	KJ434263	Hap1	KJ434286	Hap1	KJ434288
	DQ-17	Hap42	KJ434229	Hap1	KJ434263	Hap1	KJ434286	Hap1	KJ434288
	DQ-18	Hap42	KJ434229	Hap1	KJ434263	Hap1	KJ434286	Hap1	KJ434288
	DQ-19	Hap15	KJ434202	Hap1	KJ434263	Hap1	KJ434286	Hap1	KJ434288
	DQ-20	Hap15	KJ434202	Hap1	KJ434263	Hap1	KJ434286	Hap1	KJ434288
	DQ-21	Hap42	KJ434229	Hap1	KJ434263	Hap1	KJ434286	Hap1	KJ434288
	DQ-22	Hap15	KJ434202	Hap1	KJ434263	Hap1	KJ434286	Hap1	KJ434288
	DQ-23	Hap15	KJ434202	Hap1	KJ434263	Hap1	KJ434286	Hap1	KJ434288
GZ	GZ-1	Hap42	KJ434229	Hap22	KJ434284	Hap2	KJ434287	Hap4	KJ434291
	GZ-2	Hap30	KJ434217	Hap22	KJ434284	Hap2	KJ434287	Hap4	KJ434291
						Hap1	KJ434286		
	GZ-3	Hap42	KJ434229	Hap22	KJ434284	Hap2	KJ434287	Hap4	KJ434291
				Hap23	KJ434285				
	GZ-4	Hap42	KJ434229	Hap22	KJ434284	Hap2	KJ434287	Hap4	KJ434291
	GZ-5	Hap42	KJ434229	Hap22	KJ434284	Hap2	KJ434287	Hap4	KJ434291
	GZ-6	Hap42	KJ434229	Hap22	KJ434284	Hap2	KJ434287	Hap4	KJ434291
	GZ-7	Hap42	KJ434229	Hap22	KJ434284	Hap2	KJ434287	Hap4	KJ434291
	GZ-8	Hap31	KJ434218	Hap22	KJ434284	Hap2	KJ434287	Hap4	KJ434291
	GZ-9	Hap42	KJ434229	Hap22	KJ434284	Hap2	KJ434287	Hap4	KJ434291
	GZ-10	Hap30	KJ434217	Hap22	KJ434284	Hap1	KJ434286	Hap4	KJ434291
	GZ-11	Hap42	KJ434229	Hap22	KJ434284	Hap2	KJ434287	Hap4	KJ434291
	GZ-12	Hap42	KJ434229	Hap22	KJ434284	Hap2	KJ434287	Hap1	KJ434288
				Hap23	KJ434285				
	GZ-13	Hap42	KJ434229	Hap22	KJ434284	Hap2	KJ434287	Hap4	KJ434291
	GZ-14	Hap42	KJ434229	Hap22	KJ434284	Hap2	KJ434287	Hap4	KJ434291
	GZ-15	Hap42	KJ434229	Hap22	KJ434284	Hap2	KJ434287	Hap4	KJ434291
	GZ-16	Hap42	KJ434229	Hap22	KJ434284	Hap2	KJ434287	Hap4	KJ434291
	GZ-17	Hap42	KJ434229	Hap22	KJ434284	Hap2	KJ434287	Hap4	KJ434291
	GZ-18	Hap30	KJ434217	Hap22	KJ434284	Hap2	KJ434287	Hap4	KJ434291
	GZ-19	Hap42	KJ434229	Hap22	KJ434284	Hap1	KJ434286	Hap4	KJ434291
						Hap2	KJ434287		
	GZ-21	Hap42	KJ434229	Hap22	KJ434284	Hap2	KJ434287	Hap4	KJ434291
LN	LN-1	Hap68	KJ434255	Hap19	KJ434281	Hap2	KJ434287	Hap5	KJ434292
	LN-2	Hap68	KJ434255	Hap19	KJ434281	Hap2	KJ434287	Hap5	KJ434292
	LN-3	Hap68	KJ434255	Hap19	KJ434281	Hap2	KJ434287	Hap5	KJ434292
	LN-4	Hap68	KJ434255	Hap19	KJ434281	Hap2	KJ434287	Hap5	KJ434292
	LN-5	Hap68	KJ434255	Hap19	KJ434281	Hap2	KJ434287	Hap5	KJ434292
	LN-6	Hap68	KJ434255	Hap19	KJ434281	Hap2	KJ434287	Hap5	KJ434292

	LN-7	Hap68	KJ434255	Hap19	KJ434281	Hap2	KJ434287	Hap4	KJ434291
	LN-8	Hap68	KJ434255	Hap19	KJ434281	Hap2	KJ434287	Hap4	KJ434291
	LN-9	Hap68	KJ434255	Hap19	KJ434281	Hap2	KJ434287	Hap4	KJ434291
	LN-10	Hap68	KJ434255	Hap19	KJ434281	Hap2	KJ434287	Hap5	KJ434292
	LN-11	Hap68	KJ434255	Hap19	KJ434281	Hap2	KJ434287	Hap5	KJ434292
	LN-12	Hap68	KJ434255	Hap19	KJ434281	Hap2	KJ434287	Hap5	KJ434292
	LN-13	Hap68	KJ434255	Hap19	KJ434281	Hap2	KJ434287	Hap4	KJ434291
	LN-14	Hap68	KJ434255	Hap19	KJ434281	Hap2	KJ434287	Hap5	KJ434292
	LN-15	Hap68	KJ434255	Hap19	KJ434281	Hap2	KJ434287	Hap5	KJ434292
	LN-16	Hap68	KJ434255	Hap19	KJ434281	Hap2	KJ434287	Hap4	KJ434291
	LN-17	Hap68	KJ434255	Hap19	KJ434281	Hap2	KJ434287	Hap5	KJ434292
	LN-18	Hap68	KJ434255	Hap19	KJ434281	Hap2	KJ434287	Hap4	KJ434291
	LN-19	Hap68	KJ434255	Hap19	KJ434281	Hap2	KJ434287	Hap4	KJ434291
	LN-20	Hap68	KJ434255	Hap19	KJ434281	Hap2	KJ434287	-	-
LL	LL-1	Hap68	KJ434255	Hap19	KJ434281	Hap2	KJ434287	Hap4	KJ434291
	LL-2	Hap63	KJ434250	Hap19	KJ434281	Hap2	KJ434287	Hap4	KJ434291
	LL-3	Hap68	KJ434255	Hap19	KJ434281	Hap2	KJ434287	Hap4	KJ434291
	LL-4	Hap68	KJ434255	Hap19	KJ434281	Hap2	KJ434287	Hap4	KJ434291
	LL-5	Hap68	KJ434255	Hap19	KJ434281	Hap2	KJ434287	Hap4	KJ434291
	LL-6	Hap62	KJ434249	Hap19	KJ434281	Hap2	KJ434287	Hap4	KJ434291
	LL-7	Hap68	KJ434255	Hap19	KJ434281	Hap2	KJ434287	Hap4	KJ434291
	LL-8	Hap62	KJ434249	Hap19	KJ434281	Hap2	KJ434287	Hap4	KJ434291
	LL-9	Hap68	KJ434255	Hap19	KJ434281	Hap2	KJ434287	Hap4	KJ434291
	LL-10	Hap68	KJ434249	Hap19	KJ434281	Hap2	KJ434287	Hap4	KJ434291
	LL-11	Hap62	KJ434249	Hap19	KJ434281	Hap2	KJ434287	Hap4	KJ434291
	LL-12	Hap62	KJ434249	Hap19	KJ434281	Hap2	KJ434287	Hap4	KJ434291
	LL-13	Hap62	KJ434249	Hap19	KJ434281	Hap2	KJ434287	Hap4	KJ434291
	LL-14	Hap62	KJ434249	Hap19	KJ434281	Hap2	KJ434287	Hap4	KJ434291
	LL-15	Hap68	KJ434255	Hap19	KJ434281	Hap2	KJ434287	Hap4	KJ434291
	LL-16	Hap59	KJ434246	Hap19	KJ434281	Hap2	KJ434287	Hap4	KJ434291
	LL-17	Hap68	KJ434255	Hap19	KJ434281	Hap2	KJ434287	Hap4	KJ434291
	LL-18	Hap62	KJ434249	Hap19	KJ434281	Hap2	KJ434287	Hap4	KJ434291
	LL-19	Hap68	KJ434255	Hap19	KJ434281	Hap2	KJ434287	Hap4	KJ434291
	LL-20	Hap68	KJ434255	Hap19	KJ434281	Hap2	KJ434287	Hap4	KJ434291
XQ	XQ-1	Hap42	KJ434229	Hap1	KJ434263	Hap1	KJ434286	Hap1	KJ434288
	XQ-2	Hap42	KJ434229	Hap1	KJ434263	Hap1	KJ434286	Hap1	KJ434288
	XQ-3	Hap42	KJ434229	Hap1	KJ434263	Hap1	KJ434286	Hap1	KJ434288
	XQ-4	Hap42	KJ434229	Hap1	KJ434263	Hap1	KJ434286	Hap1	KJ434288
	XQ-5	Hap42	KJ434229	Hap1	KJ434263	Hap1	KJ434286	Hap1	KJ434288
	XQ-6	Hap42	KJ434229	Hap1	KJ434263	Hap1	KJ434286	Hap1	KJ434288
	XQ-7	Hap42	KJ434229	Hap1	KJ434263	Hap1	KJ434286	Hap1	KJ434288
	XQ-8	Hap42	KJ434229	Hap1	KJ434263	Hap1	KJ434286	Hap1	KJ434288

	XQ-9	Hap42	KJ434229	Hap1	KJ434263	Hap1	KJ434286	Hap1	KJ434288
	XQ-10	Hap42	KJ434229	Hap1	KJ434263	Hap1	KJ434286	Hap1	KJ434288
	XQ-11	Hap42	KJ434229	Hap1	KJ434263	Hap1	KJ434286	Hap1	KJ434288
	XQ-12	Hap42	KJ434229	Hap1	KJ434263	Hap1	KJ434286	Hap1	KJ434288
	XQ-13	Hap42	KJ434229	Hap1	KJ434263	Hap1	KJ434286	Hap1	KJ434288
	XQ-14	Hap42	KJ434229	Hap1	KJ434263	Hap1	KJ434286	Hap1	KJ434288
	XQ-15	Hap42	KJ434229	Hap1	KJ434263	Hap1	KJ434286	Hap1	KJ434288
	XQ-16	Hap42	KJ434229	Hap1	KJ434263	Hap1	KJ434286	Hap1	KJ434288
	XQ-17	Hap42	KJ434229	Hap1	KJ434263	Hap1	KJ434286	Hap1	KJ434288
	XQ-18	Hap42	KJ434229	Hap1	KJ434263	Hap1	KJ434286	Hap1	KJ434288
	XQ-19	Hap42	KJ434229	Hap1	KJ434263	Hap1	KJ434286	Hap1	KJ434288
	XQ-20	Hap42	KJ434229	Hap1	KJ434263	Hap1	KJ434286	Hap1	KJ434288
	XQ-21	Hap42	KJ434229	Hap1	KJ434263	Hap1	KJ434286	Hap1	KJ434288
	XQ-22	Hap42	KJ434229	Hap1	KJ434263	Hap1	KJ434286	Hap1	KJ434288
	XQ-23	Hap42	KJ434229	Hap1	KJ434263	Hap1	KJ434286	Hap1	KJ434288
	XQ-24	Hap42	KJ434229	Hap1	KJ434263	Hap1	KJ434286	Hap1	KJ434288
QS	QS-1	Hap49	KJ434236	Hap3	KJ434265	Hap1	KJ434286	Hap1	KJ434288
	QS-2	Hap49	KJ434236	Hap3	KJ434265	Hap1	KJ434286	Hap1	KJ434288
	QS-3	Hap49	KJ434236	Hap3	KJ434265	Hap1	KJ434286	Hap1	KJ434288
	QS-4	Hap49	KJ434236	Hap3	KJ434265	Hap1	KJ434286	Hap1	KJ434288
	QS-5	Hap49	KJ434236	Hap3	KJ434265	Hap1	KJ434286	Hap1	KJ434288
	QS-6	Hap49	KJ434236	Hap3	KJ434265	Hap1	KJ434286	Hap1	KJ434288
	QS-7	Hap49	KJ434236	Hap3	KJ434265	Hap1	KJ434286	Hap1	KJ434288
	QS-8	Hap49	KJ434236	Hap3	KJ434265	Hap1	KJ434286	Hap1	KJ434288
	QS-9	Hap49	KJ434236	Hap3	KJ434265	Hap1	KJ434286	Hap1	KJ434288
	QS-10	Hap49	KJ434236	Hap3	KJ434265	Hap1	KJ434286	Hap1	KJ434288
	QS-11	Hap49	KJ434236	Hap3	KJ434265	Hap1	KJ434286	Hap1	KJ434288
	QS-12	Hap49	KJ434236	Hap3	KJ434265	Hap1	KJ434286	Hap1	KJ434288
	QS-13	Hap49	KJ434236	Hap3	KJ434265	Hap1	KJ434286	Hap1	KJ434288
	QS-14	Hap49	KJ434236	Hap3	KJ434265	Hap1	KJ434286	Hap1	KJ434288
	QS-15	Hap49	KJ434236	Hap3	KJ434265	Hap1	KJ434286	Hap1	KJ434288
	QS-16	Hap49	KJ434236	Hap3	KJ434265	Hap1	KJ434286	Hap1	KJ434288
	QS-17	Hap48	KJ434235	Hap3	KJ434265	Hap1	KJ434286	Hap2	KJ434289
	QS-18	Hap49	KJ434236	Hap3	KJ434265	Hap1	KJ434286	Hap1	KJ434288
	QS-19	Hap49	KJ434236	Hap3	KJ434265	Hap1	KJ434286	Hap1	KJ434288
	QS-20	Hap49	KJ434236	Hap3	KJ434265	Hap1	KJ434286	Hap1	KJ434288
								Hap1	KJ434288
NG	NG-1	Hap42	KJ434229	Hap1	KJ434263	Hap1	KJ434286	Hap2	KJ434289
	NG-2	Hap42	KJ434229	Hap1	KJ434263	Hap1	KJ434286	Hap2	KJ434289

	NG-3	Hap42	KJ434229	Hap1	KJ434263	Hap1	KJ434286	Hap1	KJ434288
	NG-4	Hap42	KJ434229	Hap1	KJ434263	Hap1	KJ434286	Hap1	KJ434288
	NG-5	Hap42	KJ434229	Hap1	KJ434263	Hap1	KJ434286	Hap1	KJ434288
	NG-6	Hap42	KJ434229	Hap1	KJ434263	Hap1	KJ434286	Hap1	KJ434288
	NG-7	Hap42	KJ434229	Hap2	KJ434264	Hap1	KJ434286	Hap1	KJ434288
	NG-8	Hap42	KJ434229	Hap1	KJ434263	Hap1	KJ434286	Hap1	KJ434288
	NG-9	Hap42	KJ434229	Hap2	KJ434264	Hap1	KJ434286	Hap1	KJ434288
	NG-10	Hap42	KJ434229	Hap14	KJ434276	Hap1	KJ434286	Hap1	KJ434288
	NG-11	Hap42	KJ434229	Hap2	KJ434264	Hap1	KJ434286	Hap1	KJ434288
	NG-12	Hap42	KJ434229	Hap2	KJ434264	Hap1	KJ434286	Hap1	KJ434288
	NG-13	Hap42	KJ434229	Hap1	KJ434263	Hap1	KJ434286	Hap1	KJ434288
	NG-14	Hap42	KJ434229	Hap1	KJ434263	Hap1	KJ434286	Hap1	KJ434288
	NG-15	Hap42	KJ434229	Hap1	KJ434263	Hap1	KJ434286	Hap1	KJ434288
	NG-16	Hap42	KJ434229	Hap1	KJ434263	Hap1	KJ434286	Hap1	KJ434288
	NG-17	Hap42	KJ434229	Hap2	KJ434264	Hap1	KJ434286	Hap1	KJ434288
	NG-18	Hap42	KJ434229	Hap1	KJ434263	Hap1	KJ434286	Hap1	KJ434288
	NG-19	Hap42	KJ434229	Hap1	KJ434263	Hap1	KJ434286	Hap2	KJ434289
	NG-20	Hap42	KJ434229	Hap1	KJ434263	Hap1	KJ434286	Hap1	KJ434288
	NG-21	Hap42	KJ434229	Hap1	KJ434263	Hap1	KJ434286	Hap1	KJ434288
								Hap2	KJ434289
	NG-22	Hap21	KJ434208	Hap2	KJ434264	Hap1	KJ434286	Hap1	KJ434288
BD	BD-1	Hap38	KJ434225	Hap1	KJ434263	Hap1	KJ434286	Hap1	KJ434288
	BD-2	Hap38	KJ434225	Hap1	KJ434263	Hap1	KJ434286	Hap1	KJ434288
	BD-3	Hap38	KJ434225	Hap1	KJ434263	Hap1	KJ434286	Hap1	KJ434288
	BD-4	Hap42	KJ434229	Hap1	KJ434263	Hap1	KJ434286	Hap1	KJ434288
	BD-5	Hap38	KJ434225	Hap1	KJ434263	Hap1	KJ434286	Hap1	KJ434288
	BD-6	Hap38	KJ434225	Hap1	KJ434263	Hap1	KJ434286	Hap1	KJ434288
	BD-7	Hap38	KJ434225	Hap1	KJ434263	Hap1	KJ434286	Hap1	KJ434288
	BD-8	Hap38	KJ434225	Hap1	KJ434263	Hap1	KJ434286	Hap1	KJ434288
	BD-9	Hap42	KJ434229	Hap1	KJ434263	Hap1	KJ434286	Hap1	KJ434288
	BD-10	Hap38	KJ434225	Hap1	KJ434263	Hap1	KJ434286	Hap1	KJ434288
	BD-11	Hap42	KJ434229	Hap1	KJ434263	Hap1	KJ434286	Hap1	KJ434288
	BD-12	Hap42	KJ434229	Hap1	KJ434263	Hap1	KJ434286	Hap1	KJ434288
	BD-13	Hap38	KJ434225	Hap1	KJ434263	Hap1	KJ434286	Hap1	KJ434288
	BD-14	Hap38	KJ434225	Hap1	KJ434263	Hap1	KJ434286	Hap1	KJ434288
	BD-15	Hap42	KJ434229	Hap1	KJ434263	Hap1	KJ434286	Hap1	KJ434288
	BD-16	Hap38	KJ434225	Hap1	KJ434263	Hap1	KJ434286	Hap1	KJ434288
	BD-17	Hap42	KJ434229	Hap1	KJ434263	Hap1	KJ434286	Hap1	KJ434288
	BD-18	Hap42	KJ434229	Hap1	KJ434263	Hap1	KJ434286	Hap1	KJ434288
	BD-19	Hap38	KJ434225	Hap1	KJ434263	Hap1	KJ434286	Hap1	KJ434288
	BD-20	Hap38	KJ434225	Hap1	KJ434263	Hap1	KJ434286	Hap1	KJ434288

	BD-21	Hap38	KJ434225	Hap1	KJ434263	Hap1	KJ434286	Hap1	KJ434288
	BD-22	Hap42	KJ434229	Hap1	KJ434263	Hap1	KJ434286	Hap1	KJ434288
	BD-23	Hap42	KJ434229	Hap1	KJ434263	Hap1	KJ434286	Hap1	KJ434288
	BD-24	Hap38	KJ434225	Hap1	KJ434263	Hap1	KJ434286	Hap1	KJ434288
QG	QG-1	Hap42	KJ434229	Hap1	KJ434263	Hap1	KJ434286	Hap1	KJ434288
								Hap2	KJ434289
	QG-2	Hap42	KJ434229	Hap1	KJ434263	Hap1	KJ434286	Hap1	KJ434288
								Hap2	KJ434289
	QG-3	Hap42	KJ434229	Hap1	KJ434263	Hap1	KJ434286	Hap1	KJ434288
								Hap2	KJ434289
	QG-4	Hap23	KJ434210	Hap1	KJ434263	Hap1	KJ434286	Hap1	KJ434288
	QG-5	Hap25	KJ434212	Hap1	KJ434263	Hap1	KJ434286	Hap2	KJ434289
		Hap42	KJ434229	Hap1	KJ434263	Hap1	KJ434286	Hap1	KJ434288
	QG-6							Hap1	KJ434288
	QG-7	Hap42	KJ434229	Hap1	KJ434263	Hap1	KJ434286	Hap1	KJ434288
								Hap2	KJ434289
	QG-8	Hap42	KJ434229	Hap1	KJ434263	Hap1	KJ434286	Hap1	KJ434288
								Hap2	KJ434289
	QG-9	Hap23	KJ434210	Hap1	KJ434263	Hap1	KJ434286	Hap1	KJ434288
	QG-10	Hap42	KJ434229	Hap1	KJ434263	Hap1	KJ434286	Hap1	KJ434288
	QG-11	Hap22	KJ434209	Hap15	KJ434277	Hap1	KJ434286	Hap1	KJ434288
								Hap2	KJ434289
	QG-12	Hap42	KJ434229	Hap1	KJ434263	Hap1	KJ434286	Hap2	KJ434289
	QG-13	Hap42	KJ434229	Hap1	KJ434263	Hap1	KJ434286	Hap1	KJ434288
								Hap2	KJ434289
	QG-14	Hap42	KJ434229	Hap1	KJ434263	Hap1	KJ434286	Hap1	KJ434288
								Hap2	KJ434289
	QG-15	Hap45	KJ434232	Hap1	KJ434263	Hap1	KJ434286	Hap1	KJ434288
								Hap2	KJ434289
	QG-18	Hap42	KJ434229	Hap1	KJ434263	Hap1	KJ434286	Hap1	KJ434288
								Hap2	KJ434289

	QG-19	Hap44	KJ434231	Hap1	KJ434263	Hap1	KJ434286	Hap1	KJ434288
								Hap2	KJ434289
	QG-23	Hap42	KJ434229	Hap1	KJ434263	Hap1	KJ434286	Hap1	KJ434288
	QG-24	Hap7	KJ434194	Hap1	KJ434263	Hap1	KJ434286	Hap1	KJ434288
	QG-26	Hap24	KJ434211	Hap1	KJ434263	Hap1	KJ434286	Hap1	KJ434288
								Hap2	KJ434289
CN	CN-1	Hap47	KJ434234	Hap1	KJ434263	Hap1	KJ434286	Hap1	KJ434288
								Hap3	KJ434290
	CN-2	Hap50	KJ434237	Hap15	KJ434277	Hap1	KJ434286	Hap1	KJ434288
		Hap49		Hap18	KJ434280				
	CN-3	Hap47	KJ434234	Hap1	KJ434263	Hap1	KJ434286	Hap1	KJ434288
								Hap2	KJ434289
	CN-4	Hap42	KJ434229	Hap1	KJ434263	Hap1	KJ434286	Hap1	KJ434288
		Hap49		Hap3	KJ434265			Hap2	KJ434289
	CN-5	Hap49	KJ434236	Hap1	KJ434263	Hap1	KJ434286	Hap1	KJ434288
	CN-6	Hap49	KJ434236	Hap1	KJ434263	Hap1	KJ434286	Hap1	KJ434288
								Hap2	KJ434289
	CN-7	Hap47	KJ434234	Hap1	KJ434263	Hap1	KJ434286	Hap1	KJ434288
	CN-8	Hap49	KJ434236	Hap1	KJ434263	Hap1	KJ434286	Hap1	KJ434288
	CN-9	Hap46	KJ434233	Hap15	KJ434277	Hap1	KJ434286	Hap1	KJ434288
		Hap49		Hap18	KJ434280				
	CN-10	Hap49	KJ434236	Hap1	KJ434263	Hap1	KJ434286	Hap1	KJ434288
		Hap49		Hap3	KJ434265				
	CN-11	Hap51	KJ434238	Hap3	KJ434265	Hap1	KJ434286	Hap1	KJ434288
	CN-12	Hap49	KJ434236	Hap1	KJ434263	Hap1	KJ434286	Hap1	KJ434288
	CN-13	Hap47	KJ434234	Hap1	KJ434263	Hap1	KJ434286	Hap1	KJ434288
								Hap2	KJ434289
	CN-14	Hap49	KJ434236	Hap1	KJ434263	Hap1	KJ434286	Hap1	KJ434288
	CN-15	Hap51	KJ434238	Hap1	KJ434263	Hap1	KJ434286	Hap1	KJ434288
	CN-16	Hap49	KJ434236	Hap1	KJ434263	Hap1	KJ434286	Hap1	KJ434288
	CN-17	Hap49	KJ434236	Hap1	KJ434263	Hap1	KJ434286	Hap1	KJ434288
	CN-18	Hap42	KJ434229	Hap1	KJ434263	Hap1	KJ434286	Hap1	KJ434288
								Hap3	KJ434290
	CN-19	Hap49	KJ434236	Hap1	KJ434263	Hap1	KJ434286	-	-
				Hap3	KJ434265				
	CN-20	Hap18	KJ434205	Hap1	KJ434263	Hap1	KJ434286	Hap1	KJ434288
								Hap2	

									KJ434289
CD	CD-1	Hap42	KJ434229	Hap2	KJ434264	Hap1	KJ434286	Hap1	KJ434288
	CD-2	Hap17	KJ434204	Hap2	KJ434264	Hap1	KJ434286	Hap1	KJ434288
	CD-3	Hap42	KJ434229	Hap2	KJ434264	Hap1	KJ434286	Hap1	KJ434288
	CD-4	Hap42	KJ434229	Hap24	KJ810612	Hap1	KJ434286	Hap1	KJ434288
				Hap4	KJ434266				
	CD-5	Hap20	KJ434207	Hap2	KJ434264	Hap1	KJ434286	Hap1	KJ434288
	CD-6	Hap42	KJ434229	Hap2	KJ434264	Hap1	KJ434286	Hap1	KJ434288
	CD-7	Hap42	KJ434229	Hap2	KJ434264	Hap1	KJ434286	Hap1	KJ434288
	CD-8	Hap20	KJ434207	Hap2	KJ434264	Hap1	KJ434286	Hap1	KJ434288
	CD-9	Hap42	KJ434229	Hap2	KJ434264	Hap1	KJ434286	Hap1	KJ434288
	CD-10	Hap42	KJ434229	Hap2	KJ434264	Hap1	KJ434286	Hap1	KJ434288
	CD-11	Hap42	KJ434229	Hap2	KJ434264	Hap1	KJ434286	Hap1	KJ434288
	CD-12	Hap42	KJ434229	Hap2	KJ434264	Hap1	KJ434286	Hap1	KJ434288
	CD-13	Hap42	KJ434229	Hap2	KJ434264	Hap1	KJ434286	Hap1	KJ434288
	CD-14	Hap42	KJ434229	Hap24	KJ810612	Hap1	KJ434286	Hap1	KJ434288
				Hap4	KJ434266				
	CD-15	Hap42	KJ434229	Hap2	KJ434264	Hap1	KJ434286	Hap1	KJ434288
	CD-16	Hap42	KJ434229	Hap2	KJ434264	Hap1	KJ434286	Hap1	KJ434288
	CD-17	Hap42	KJ434229	Hap2	KJ434264	Hap1	KJ434286	Hap1	KJ434288
	CD-18	Hap42	KJ434229	Hap24	KJ810612	Hap1	KJ434286	Hap1	KJ434288
				Hap4	KJ434266				
	CD-19	Hap65	KJ434252	-	-	Hap1	KJ434286	Hap1	KJ434288
	CD-20	Hap20	KJ434207	Hap2	KJ434264	Hap1	KJ434286	Hap1	KJ434288
PL	PL-1	Hap20	KJ434207	Hap22	KJ434284	Hap2	KJ434287	Hap4	KJ434291
	PL-2	Hap68	KJ434255	Hap22	KJ434284	Hap2	KJ434287	Hap4	KJ434291
	PL-3	Hap68	KJ434255	Hap22	KJ434284	Hap2	KJ434287	Hap4	KJ434291
	PL-4	Hap68	KJ434255	Hap22	KJ434284	Hap2	KJ434287	Hap4	KJ434291
	PL-5	Hap68	KJ434255	Hap22	KJ434284	Hap2	KJ434287	Hap4	KJ434291
	PL-6	Hap68	KJ434255	Hap22	KJ434284	Hap2	KJ434287	Hap4	KJ434291
	PL-7	Hap68	KJ434255	Hap22	KJ434284	Hap2	KJ434287	Hap4	KJ434291
	PL-8	Hap68	KJ434255	Hap22	KJ434284	Hap2	KJ434287	Hap4	KJ434291
	PL-9	Hap68	KJ434255	Hap22	KJ434284	Hap2	KJ434287	Hap4	KJ434291
	PL-10	Hap68	KJ434255	Hap22	KJ434284	Hap2	KJ434287	Hap4	KJ434291
	PL-11	Hap68	KJ434255	Hap22	KJ434284	Hap2	KJ434287	Hap4	KJ434291
	PL-12	Hap68	KJ434255	Hap22	KJ434284	Hap2	KJ434287	Hap4	KJ434291
	PL-13	Hap68	KJ434255	Hap22	KJ434284	Hap2	KJ434287	Hap4	KJ434291
	PL-14	Hap68	KJ434255	Hap22	KJ434284	Hap2	KJ434287	Hap4	KJ434291
	PL-15	Hap68	KJ434255	Hap22	KJ434284	Hap2	KJ434287	Hap4	KJ434291
	PL-16	Hap68	KJ434255	Hap22	KJ434284	Hap2	KJ434287	Hap4	KJ434291
	PL-17	Hap68	KJ434255	Hap22	KJ434284	Hap2	KJ434287	Hap4	KJ434291
	PL-18	Hap68	KJ434255	Hap22	KJ434284	Hap2	KJ434287	Hap4	KJ434291

	PL-19	Hap68	KJ434255	Hap22	KJ434284	Hap2	KJ434287	Hap4	KJ434291
	PL-20	Hap68	KJ434255	Hap22	KJ434284	Hap2	KJ434287	Hap4	KJ434291
DX	DX-1	Hap6	KJ434193	Hap1	KJ434263	Hap1	KJ434286	Hap1	KJ434288
	DX-2	Hap19	KJ434206	Hap1	KJ434263	Hap1	KJ434286	Hap1	KJ434288
	DX-3	Hap42	KJ434229	Hap1	KJ434263	Hap1	KJ434286	Hap1	KJ434288
	DX-4	Hap42	KJ434229	Hap1	KJ434263	Hap1	KJ434286	Hap1	KJ434288
	DX-5	Hap42	KJ434229	Hap1	KJ434263	Hap1	KJ434286	Hap1	KJ434288
	DX-6	Hap42	KJ434229	Hap1	KJ434263	Hap1	KJ434286	Hap1	KJ434288
	DX-7	Hap42	KJ434229	Hap1	KJ434263	Hap1	KJ434286	Hap1	KJ434288
	DX-8	Hap42	KJ434229	Hap1	KJ434263	Hap1	KJ434286	Hap1	KJ434288
	DX-9	Hap42	KJ434229	Hap1	KJ434263	Hap1	KJ434286	Hap1	KJ434288
	DX-10	Hap42	KJ434229	Hap1	KJ434263	Hap1	KJ434286	Hap1	KJ434288
	DX-11	Hap42	KJ434229	Hap1	KJ434263	Hap1	KJ434286	Hap1	KJ434288
	DX-12	Hap42	KJ434229	Hap1	KJ434263	Hap1	KJ434286	Hap1	KJ434288
	DX-13	Hap42	KJ434229	Hap1	KJ434263	Hap1	KJ434286	Hap1	KJ434288
	DX-14	Hap73	KJ434260	Hap1	KJ434263	Hap1	KJ434286	Hap1	KJ434288
	DX-15	Hap42	KJ434229	Hap1	KJ434263	Hap1	KJ434286	Hap1	KJ434288
	DX-16	Hap42	KJ434229	Hap1	KJ434263	Hap1	KJ434286	Hap1	KJ434288
	DX-17	Hap42	KJ434229	Hap1	KJ434263	Hap1	KJ434286	-	-
	DX-18	Hap42	KJ434229	Hap1	KJ434263	Hap1	KJ434286	Hap1	KJ434288
	DX-19	Hap42	KJ434229	Hap1	KJ434263	Hap1	KJ434286	Hap1	KJ434288
	DX-20	Hap42	KJ434229	Hap1	KJ434263	Hap1	KJ434286	Hap1	KJ434288
	DX-21	Hap42	KJ434229	Hap1	KJ434263	Hap1	KJ434286	Hap1	KJ434288
YB	YB-1	Hap13	KJ434200	Hap1	KJ434263	Hap1	KJ434286	Hap1	KJ434288
	YB-2	Hap13	KJ434200	Hap2	KJ434264	Hap1	KJ434286	Hap1	KJ434288
	YB-3	Hap13	KJ434200	Hap15	KJ434277	Hap1	KJ434286	Hap1	KJ434288
	YB-4	Hap14	KJ434201	Hap1	KJ434263	Hap1	KJ434286	Hap1	KJ434288
	YB-5	Hap14	KJ434201	Hap2	KJ434264	Hap1	KJ434286	Hap1	KJ434288
	YB-6	Hap13	KJ434200	Hap2	KJ434264	Hap1	KJ434286	Hap1	KJ434288
	YB-7	Hap13	KJ434200	Hap2	KJ434264	Hap1	KJ434286	Hap1	KJ434288
	YB-8	Hap13	KJ434200	Hap2	KJ434264	Hap1	KJ434286	Hap1	KJ434288
	YB-9	Hap13	KJ434200	Hap15	KJ434277	Hap1	KJ434286	Hap1	KJ434288
	YB-10	Hap13	KJ434200	Hap1	KJ434263	Hap1	KJ434286	Hap1	KJ434288
	YB-11	Hap13	KJ434200	Hap2	KJ434264	Hap1	KJ434286	Hap1	KJ434288
	YB-12	Hap13	KJ434200	Hap2	KJ434264	Hap1	KJ434286	-	
						Hap2	KJ434287		
	YB-13	Hap74	KJ434261	Hap2	KJ434264	Hap1	KJ434286	Hap1	KJ434288
	YB-14	Hap13	KJ434200	Hap2	KJ434264	Hap1	KJ434286	Hap1	KJ434288
	YB-15	Hap13	KJ434200	Hap1	KJ434263	Hap1	KJ434286	Hap1	KJ434288
	YB-16	Hap13	KJ434200	Hap2	KJ434264	Hap1	KJ434286	Hap1	KJ434288
	YB-17	Hap13	KJ434200	Hap2	KJ434264	Hap1	KJ434286	Hap1	KJ434288
	YB-18	Hap13	KJ434200	Hap2	KJ434264	Hap1	KJ434286	Hap1	KJ434288

	YB-19	Hap13	KJ434200	Hap2	KJ434264	Hap1	KJ434286	Hap1	KJ434288
	YB-20	Hap13	KJ434200	Hap2	KJ434264	Hap1	KJ434286	Hap1	KJ434288
	YB-21	Hap75	KJ434262	Hap2	KJ434264	Hap1	KJ434286	Hap1	KJ434288
						Hap2	KJ434287		
	YB-22	Hap13	KJ434200	Hap1	KJ434263	Hap1	KJ434286	Hap1	KJ434288
	YB-23	Hap13	KJ434200	Hap2	KJ434264	Hap1	KJ434286	Hap1	KJ434288
	YB-24	Hap13	KJ434200	Hap2	KJ434264	Hap1	KJ434286	Hap1	KJ434288
	YB-25	Hap13	KJ434200	Hap2	KJ434264	Hap1	KJ434286	Hap1	KJ434288
	YB-26	Hap13	KJ434200	Hap2	KJ434264	Hap1	KJ434286	Hap1	KJ434288
BJ	BJ-1	Hap42	KJ434229	Hap8	KJ434270	Hap1	KJ434286	Hap1	KJ434288
	BJ-2	Hap42	KJ434229	Hap1	KJ434263	Hap1	KJ434286	Hap1	KJ434288
				Hap8	KJ434270			Hap2	KJ434289
	BJ-3	Hap42	KJ434229	Hap1	KJ434263	Hap1	KJ434286	Hap1	KJ434288
				Hap8	KJ434270				
	BJ-4	Hap53	KJ434240	Hap1	KJ434263	Hap1	KJ434286	Hap1	KJ434288
				Hap8	KJ434270				
	BJ-5	Hap42	KJ434229	Hap15	KJ434277	Hap1	KJ434286	Hap1	KJ434288
				Hap16	KJ434278				
	BJ-6	Hap42	KJ434229	Hap8	KJ434270	Hap1	KJ434286	Hap1	KJ434288
XH	XH-1	Hap68	KJ434255	Hap19	KJ434281	Hap2	KJ434287	Hap4	KJ434291
	XH-2	Hap68	KJ434255	Hap19	KJ434281	Hap2	KJ434287	Hap4	KJ434291
LU	LU-1	Hap5	KJ434192	Hap3	KJ434265	Hap1	KJ434286	Hap1	KJ434288
	LU-2	Hap1	KJ434188	Hap3	KJ434265	Hap1	KJ434286	Hap1	KJ434288
	LU-3	Hap5	KJ434192	Hap3	KJ434265	Hap1	KJ434286	Hap1	KJ434288
	LU-4	Hap5	KJ434192	Hap3	KJ434265	Hap1	KJ434286	Hap1	KJ434288
	LU-5	Hap1	KJ434188	Hap3	KJ434265	Hap1	KJ434286	Hap1	KJ434288
	LU-6	Hap1	KJ434188	Hap3	KJ434265	Hap1	KJ434286	Hap1	KJ434288
	LU-7	Hap5	KJ434192	Hap3	KJ434265	Hap1	KJ434286	Hap1	KJ434288
	LU-8	Hap5	KJ434192	Hap3	KJ434265	Hap1	KJ434286	Hap1	KJ434288
	LU-9	Hap1	KJ434188	Hap15	KJ434277	Hap1	KJ434286	Hap1	KJ434288
				Hap18	KJ434280				
	LU-10	Hap2	KJ434189	Hap3	KJ434265	Hap1	KJ434286	Hap1	KJ434288
	LU-11	Hap5	KJ434192	Hap3	KJ434265	Hap1	KJ434286	Hap1	KJ434288
	LU-12	Hap4	KJ434191	Hap15	KJ434277	Hap1	KJ434286	Hap1	KJ434288
				Hap18	KJ434280				
	LU-13	Hap2	KJ434189	Hap3	KJ434265	Hap1	KJ434286	Hap1	KJ434288
	LU-14	Hap2	KJ434189	Hap3	KJ434265	Hap1	KJ434286	Hap1	KJ434288
								Hap2	KJ434289
	LU-15	Hap5	KJ434192	Hap3	KJ434265	Hap1	KJ434286	Hap1	KJ434288

	LU-16	Hap5	KJ434192	Hap15	KJ434277	Hap1	KJ434286	Hap1	KJ434288
				Hap18	KJ434280				
	LU-17	Hap3	KJ434190	Hap1	KJ434263	Hap1	KJ434286	Hap1	KJ434288
				Hap3	KJ434265				
	LU-18	Hap1	KJ434188	Hap3	KJ434265	Hap1	KJ434286	Hap1	KJ434288
	LU-19	Hap2	KJ434189	Hap3	KJ434265	Hap1	KJ434286	Hap1	KJ434288
	LU-20	Hap1	KJ434188	Hap3	KJ434265	Hap1	KJ434286	Hap1	KJ434288
SP	SP-1	Hap9	KJ434196	Hap1	KJ434263	Hap1	KJ434286	Hap2	KJ434289
	SP-2	Hap9	KJ434196	Hap15	KJ434277	Hap1	KJ434286	Hap2	KJ434289
				Hap16	KJ434278				
	SP-3	Hap43	KJ434230	Hap15	KJ434277	Hap1	KJ434286	Hap1	KJ434288
	SP-4	Hap43	KJ434230	Hap1	KJ434263	Hap1	KJ434286	Hap1	KJ434288
				Hap8	KJ434270			Hap2	KJ434289
	SP-5	Hap52	KJ434239	Hap11	KJ434273	Hap1	KJ434286	Hap1	KJ434288
				Hap9	KJ434271				
	SP-6	Hap10	KJ434197	Hap2	KJ434264	Hap1	KJ434286	Hap1	KJ434288
								Hap2	KJ434289
	SP-7	Hap9	KJ434196	Hap15	KJ434277	Hap1	KJ434286	Hap1	KJ434288
	SP-8	Hap9	KJ434196	Hap1	KJ434263	Hap1	KJ434286	Hap1	KJ434288
								Hap2	KJ434289
	SP-9	Hap9	KJ434196	Hap1	KJ434263	Hap1	KJ434286	Hap1	KJ434288
	SP-10	Hap10	KJ434197	Hap1	KJ434263	Hap1	KJ434286	Hap1	KJ434288
								Hap2	KJ434289
	SP-11	Hap42	KJ434229	Hap11	KJ434273	Hap1	KJ434286	Hap1	KJ434288
				Hap9	KJ434271			Hap2	KJ434289
	SP-12	Hap10	KJ434197	Hap1	KJ434263	Hap1	KJ434286	Hap1	KJ434288
	SP-13	Hap42	KJ434229	Hap15	KJ434277	Hap1	KJ434286	Hap1	KJ434288
				Hap16	KJ434278			Hap2	KJ434289
	SP-14	Hap9	KJ434196	Hap1	KJ434263	Hap1	KJ434286	Hap1	KJ434288
								Hap2	KJ434289
NY	NY-1	Hap56	KJ434243	Hap15	KJ434277	Hap1	KJ434286	Hap1	KJ434288
				Hap16	KJ434278				
	NY-2	Hap52	KJ434239	Hap15	KJ434277	Hap1	KJ434286	Hap1	KJ434288

	NY-3	Hap57	KJ434244	Hap16	KJ434278	Hap1	KJ434286	Hap1	KJ434288
				Hap17	KJ434279				
	NY-4	Hap55	KJ434242	Hap16	KJ434278	Hap1	KJ434286	Hap1	KJ434288
				Hap17	KJ434279				
	NY-5	Hap55	KJ434242	Hap15	KJ434277	Hap1	KJ434286	Hap1	KJ434288
	NY-6	Hap42	KJ434229	Hap15	KJ434277	Hap1	KJ434286	Hap1	KJ434288
		Hap42		Hap16	KJ434278				
	NY-7		KJ434229	Hap16	KJ434278	Hap1	KJ434286	Hap1	KJ434288
				Hap17	KJ434279				
	NY-8	Hap55	KJ434242	Hap15	KJ434277	Hap1	KJ434286	Hap1	KJ434288
				Hap17	KJ434279				
	NY-9	Hap42	KJ434229	Hap15	KJ434277	Hap1	KJ434286	Hap1	KJ434288
				Hap16	KJ434278				
	NY-10	Hap55	KJ434242	Hap15	KJ434277	Hap1	KJ434286	Hap1	KJ434288
				Hap16	KJ434278				
	NY-11	Hap56	KJ434243	Hap15	KJ434277	Hap1	KJ434286	Hap1	KJ434288
	NY-12	Hap56	KJ434243	Hap16	KJ434278	Hap1	KJ434286	Hap1	KJ434288
	NY-13	Hap42	KJ434229	Hap16	KJ434278	Hap1	KJ434286	Hap1	KJ434288
				Hap17	KJ434279				
	NY-14	Hap54	KJ434241	Hap15	KJ434277	Hap1	KJ434286	Hap1	KJ434288
				Hap16	KJ434278				
BY	BY-1	Hap42	KJ434229	Hap1	KJ434263	Hap1	KJ434286	Hap1	KJ434288
				Hap8	KJ434270				
	BY-2	Hap42	KJ434229	Hap13	KJ434275	Hap1	KJ434286	Hap1	KJ434288
				Hap12	KJ434274				
	BY-3	Hap42	KJ434229	Hap8	KJ434270	Hap1	KJ434286	Hap1	KJ434288
								Hap2	KJ434289
	BY-4	Hap42	KJ434229	Hap2	KJ434264	Hap1	KJ434286	Hap1	KJ434288
	BY-5	Hap42	KJ434229	Hap1	KJ434263	Hap1	KJ434286	Hap1	KJ434288
				Hap8	KJ434270			Hap2	KJ434289
	BY-6	Hap42	KJ434229	Hap2	KJ434264	Hap1	KJ434286	Hap1	KJ434288
								Hap2	KJ434289
	BY-7	Hap42	KJ434229	Hap2	KJ434264	Hap1	KJ434286	Hap1	KJ434288
	BY-8	Hap42	KJ434229	Hap2	KJ434264	Hap1	KJ434286	Hap1	KJ434288
	BY-9	Hap41	KJ434228	Hap2	KJ434264	Hap1	KJ434286	Hap1	KJ434288
								Hap2	KJ434289
	BY-10	Hap42	KJ434229	Hap2	KJ434264	Hap1	KJ434286	Hap1	KJ434288
								Hap2	KJ434289

	BY-11	Hap41	KJ434228	Hap1	KJ434263	Hap1	KJ434286	Hap1	KJ434288
				Hap8	KJ434270			Hap2	KJ434289
	BY-12	Hap42	KJ434229	Hap1	KJ434263	Hap1	KJ434286	Hap1	KJ434288
				Hap8	KJ434270				
	BY-13	Hap40	KJ434227	Hap10	KJ434272	Hap1	KJ434286	Hap1	KJ434288
				Hap11	KJ434273				
	BY-14	Hap42	KJ434229	Hap2	KJ434264	Hap1	KJ434286	Hap1	KJ434288
	BY-15	Hap42	KJ434229	Hap2	KJ434264	Hap1	KJ434286	Hap1	KJ434288
								Hap2	KJ434289
	BY-16	Hap40	KJ434227	Hap10	KJ434272	Hap1	KJ434286	Hap1	KJ434288
				Hap11	KJ434273				
	BY-17	Hap42	KJ434229	Hap2	KJ434264	Hap1	KJ434286	Hap1	KJ434288
								Hap2	KJ434289
	BY-18	Hap42	KJ434229	Hap1	KJ434263	Hap1	KJ434286	Hap1	KJ434288
				Hap8	KJ434270				
	BY-19	Hap42	KJ434229	Hap1	KJ434263	Hap1	KJ434286	Hap1	KJ434288
				Hap8	KJ434270				
	BY-20	Hap42	KJ434229	Hap1	KJ434263			Hap1	KJ434288
				Hap8	KJ434270	Hap1	KJ434286		
RC	RC-1	Hap68	KJ434255	Hap19	KJ434281	Hap2	KJ434287	Hap4	KJ434291
	RC-2	Hap68	KJ434255	Hap19	KJ434281	Hap2	KJ434287	Hap4	KJ434291
	RC-3	Hap68	KJ434255	Hap19	KJ434281	Hap2	KJ434287	Hap4	KJ434291
	RC-4	Hap68	KJ434255	Hap19	KJ434281	Hap2	KJ434287	Hap4	KJ434291
	RC-5	Hap66	KJ434253	Hap19	KJ434281	Hap2	KJ434287	Hap4	KJ434291
	RC-6	Hap68	KJ434255	Hap19	KJ434281	Hap2	KJ434287	Hap4	KJ434291
	RC-7	Hap68	KJ434255	Hap19	KJ434281	Hap2	KJ434287	Hap4	KJ434291
NM	NM-1	Hap68	KJ434255	Hap22	KJ434284	Hap2	KJ434287	Hap4	KJ434291
				Hap21	KJ434283				
	NM-2	Hap69	KJ434256	Hap22	KJ434284	Hap2	KJ434287	Hap4	KJ434291
				Hap21	KJ434283				
	NM-3	Hap69	KJ434256	Hap21	KJ434283	Hap2	KJ434287	Hap4	KJ434291
	NM-4	Hap69	KJ434256	Hap21	KJ434283	Hap2	KJ434287	Hap4	KJ434291
	NM-5	Hap61	KJ434248	Hap21	KJ434283	Hap2	KJ434287	Hap4	KJ434291
	NM-6	Hap58	KJ434245	Hap21	KJ434283	Hap2	KJ434287	Hap4	KJ434291
	NM-7	Hap71	KJ434258	Hap21	KJ434283	Hap2	KJ434287	Hap4	KJ434291
	NM-8	Hap68	KJ434255	Hap21	KJ434283	Hap2	KJ434287	Hap4	KJ434291
	NM-9	Hap60	KJ434247	hap22	KJ434284	Hap2	KJ434287	Hap4	KJ434291
	NM-10	Hap69	KJ434256	Hap22	KJ434284	Hap2	KJ434287	Hap4	KJ434291
	NM-11	Hap69	KJ434256	Hap21	KJ434283	Hap2	KJ434287	Hap4	KJ434291

	NM-12	Hap68	KJ434255	Hap21	KJ434283	Hap2	KJ434287	Hap4	KJ434291
	NM-13	Hap72	KJ434259	Hap21	KJ434283	Hap2	KJ434287	Hap4	KJ434291
	NM-14	Hap70	KJ434257	Hap21	KJ434283	Hap2	KJ434287	Hap4	KJ434291
	NM-15	Hap69	KJ434256	Hap21	KJ434283	Hap2	KJ434287	Hap4	KJ434291
NT	NT-1	Hap42	KJ434229	Hap1	KJ434263	Hap1	KJ434286	Hap1	KJ434288
	NT-2	Hap42	KJ434229	Hap1	KJ434263	Hap1	KJ434286	Hap1	KJ434288
	NT-3	Hap42	KJ434229	Hap1	KJ434263	Hap1	KJ434286	Hap1	KJ434288
	NT-4	Hap42	KJ434229	Hap1	KJ434263	Hap1	KJ434286	Hap1	KJ434288
	NT-5	Hap42	KJ434229	Hap1	KJ434263	Hap1	KJ434286	Hap1	KJ434288
	NT-6	Hap42	KJ434229	Hap1	KJ434263	Hap1	KJ434286	Hap1	KJ434288
	NT-7	Hap42	KJ434229	Hap1	KJ434263	Hap1	KJ434286	Hap1	KJ434288
	NT-8	Hap42	KJ434229	Hap1	KJ434263	Hap1	KJ434286	Hap1	KJ434288
	NT-9	Hap42	KJ434229	Hap1	KJ434263	Hap1	KJ434286	Hap1	KJ434288
	NT-10	Hap42	KJ434229	Hap1	KJ434263	Hap1	KJ434286	Hap1	KJ434288
	NT-11	Hap42	KJ434229	Hap1	KJ434263	Hap1	KJ434286	Hap1	KJ434288
BQ	BQ-1	Hap42	KJ434229	Hap1	KJ434263	Hap1	KJ434286	Hap1	KJ434288
	BQ-2	Hap42	KJ434229	Hap1	KJ434263	Hap1	KJ434286	Hap1	KJ434288
	BQ-3	Hap42	KJ434229	Hap1	KJ434263	Hap1	KJ434286	Hap1	KJ434288
	BQ-4	Hap42	KJ434229	Hap1	KJ434263	Hap1	KJ434286	Hap1	KJ434288
	BQ-5	Hap42	KJ434229	Hap1	KJ434263	Hap1	KJ434286	Hap1	KJ434288
	BQ-6	Hap42	KJ434229	Hap1	KJ434263	Hap1	KJ434286	Hap1	KJ434288
	BQ-7	Hap42	KJ434229	Hap1	KJ434263	Hap1	KJ434286	Hap1	KJ434288
	BQ-8	Hap39	KJ434226	-	-	Hap1	KJ434286	Hap1	KJ434288
	BQ-9	Hap42	KJ434229	Hap1	KJ434263	Hap1	KJ434286	Hap1	KJ434288
	BQ-10	Hap42	KJ434229	Hap1	KJ434263	Hap1	KJ434286	Hap1	KJ434288
	BQ-11	Hap42	KJ434229	Hap1	KJ434263	Hap1	KJ434286	Hap1	KJ434288
	BQ-12	Hap42	KJ434229	Hap1	KJ434263	Hap1	KJ434286	Hap1	KJ434288
	BQ-13	Hap42	KJ434229	Hap1	KJ434263	Hap1	KJ434286	Hap1	KJ434288
	BQ-14	Hap42	KJ434229	Hap1	KJ434263	Hap1	KJ434286	Hap1	KJ434288
	BQ-15	Hap42	KJ434229	Hap1	KJ434263	Hap1	KJ434286	Hap1	KJ434288
RD	RD-1	Hap42	KJ434229	Hap1	KJ434263	Hap1	KJ434286	Hap1	KJ434288
	RD-2	Hap42	KJ434229	Hap5<br>Hap7	KJ434267<br>KJ434269	-	-	Hap1	KJ434288
	RD-3	Hap42	KJ434229	Hap5<br>Hap7	KJ434267<br>KJ434269	Hap1	KJ434286	Hap1	KJ434288
	RD-4	Hap42	KJ434229	Hap7	KJ434269	Hap1	KJ434286	Hap1	KJ434288
	RD-5	Hap42	KJ434229	Hap5<br>Hap7	KJ434267<br>KJ434269	Hap1	KJ434286	Hap1	KJ434288
	RD-6	Hap42	KJ434229	Hap5<br>Hap7	KJ434267<br>KJ434269	Hap1	KJ434286	Hap1	KJ434288
LC	LC-1	Hap68	KJ434255	Hap19	KJ434281	Hap2	KJ434287	Hap4	KJ434291

	LC-2	Hap68	KJ434255	Hap19	KJ434281	Hap2	KJ434287	Hap4	KJ434291
				Hap20	KJ434282				
	LC-3	Hap68	KJ434255	Hap19	KJ434281	Hap2	KJ434287	Hap4	KJ434291
	LC-4	Hap68	KJ434255	Hap19	KJ434281	Hap2	KJ434287	Hap4	KJ434291
	LC-5	Hap68	KJ434255	Hap19	KJ434281	Hap2	KJ434287	Hap4	KJ434291
	LC-6	Hap67	KJ434254	Hap19	KJ434281	Hap2	KJ434287	Hap4	KJ434291
	LC-7	Hap68	KJ434255	Hap19	KJ434281	Hap2	KJ434287	Hap4	KJ434291
				Hap20	KJ434282				
	LC-8	Hap68	KJ434255	Hap19	KJ434281	Hap2	KJ434287	Hap4	KJ434291
	LC-9	Hap68	KJ434255	Hap19	KJ434281	Hap2	KJ434287	Hap4	KJ434291
	LC-10	Hap68	KJ434255	Hap19	KJ434281	Hap2	KJ434287	Hap4	KJ434291
	LC-11	Hap68	KJ434255	Hap19	KJ434281	Hap2	KJ434287	Hap4	KJ434291
	LC-12	Hap68	KJ434255	Hap19	KJ434281	Hap2	KJ434287	Hap4	KJ434291
	LC-13	Hap68	KJ434255	Hap19	KJ434281	Hap2	KJ434287	Hap4	KJ434291
	LC-14	Hap68	KJ434255	Hap19	KJ434281	Hap2	KJ434287	Hap4	KJ434291
	LC-15	Hap68	KJ434255	Hap19	KJ434281	Hap2	KJ434287	Hap4	KJ434291
	LC-16	Hap68	KJ434255	Hap19	KJ434281	Hap2	KJ434287	Hap4	KJ434291
	LC-17	Hap68	KJ434255	Hap19	KJ434281	Hap2	KJ434287	Hap4	KJ434291
CM	CM-1	Hap42	KJ434229	Hap2	KJ434264	Hap1	KJ434286	Hap1	KJ434288
	CM-2	Hap36	KJ434223	Hap2	KJ434264	-	-	Hap1	KJ434288
	CM-3	Hap42	KJ434229	Hap5	KJ434267	Hap1	KJ434286	Hap1	KJ434288
				Hap6	KJ434268				
	CM-4	Hap42	KJ434229	Hap1	KJ434263	Hap1	KJ434286	Hap1	KJ434288
	CM-5	Hap34	KJ434221	Hap15	KJ434277	Hap1	KJ434286	Hap1	KJ434288
	CM-6	Hap42	KJ434229	Hap5	KJ434267	Hap1	KJ434286	Hap1	KJ434288
				Hap6	KJ434268				
	CM-7	Hap42	KJ434229	Hap1	KJ434263	Hap1	KJ434286	Hap1	KJ434288
	CM-8	Hap42	KJ434229	Hap5	KJ434267	Hap1	KJ434286	Hap1	KJ434288
				Hap6	KJ434268				
	CM-9	Hap42	KJ434229	Hap1	KJ434263	Hap1	KJ434286	Hap1	KJ434288
	CM-10	Hap42	KJ434229	Hap7	KJ434269	Hap1	KJ434286	Hap1	KJ434288
	CM-11	Hap33	KJ434220	Hap1	KJ434263	Hap1	KJ434286	Hap1	KJ434288
	CM-12	Hap42	KJ434229	Hap1	KJ434263	Hap1	KJ434286	Hap1	KJ434288
	CM-13	Hap35	KJ434222	Hap5	KJ434267	Hap1	KJ434286	Hap1	KJ434288
				Hap6	KJ434268				
	CM-14	Hap42	KJ434229	Hap1	KJ434263	Hap1	KJ434286	Hap1	KJ434288
	CM-15	Hap32	KJ434219	Hap1	KJ434263	Hap1	KJ434286	Hap1	KJ434288
	CM-16	Hap42	KJ434229	Hap1	KJ434263	Hap1	KJ434286	Hap1	KJ434288
	CM-17	Hap42	KJ434229	Hap1	KJ434263	Hap1	KJ434286	Hap1	KJ434288
	CM-18	Hap8	KJ434195	Hap1	KJ434263	Hap1	KJ434286	Hap1	KJ434288
	CM-19	Hap42	KJ434229	Hap1	KJ434263	Hap1	KJ434286	Hap1	KJ434288
	CM-20	Hap42	KJ434229	Hap1	KJ434263	Hap1	KJ434286	Hap1	KJ434288

Table S2 Cyt*b* haplotype frequency, nucleotide diversity (π) and haplotype diversity (*h*) with standard deviation (s.d.) in each population. Haplotypes in bold are those shared between different populations, and the rest are the private haplotypes. See Table 1 for population abbreviations.

Population	Cyt*b* clade	Haplotype (frequency)	*π*	*h* (s.d.)
BQ	E1	Hap39 (1); **Hap42** (14)	0.00013	0.133 (0.112)
BD	E1	Hap38 (15); **Hap42** (9)	0.00047	0.489 (0.057)
BY	E1	Hap40 (2); Hap41 (2); **Hap42** (16)	0.00036	0.358 (0.126)
DQ	E1	Hap15 (14); Hap16 (1); **Hap42** (8)	0.00056	0.530 (0.071)
DX	E1	Hap6 (1); Hap19 (1); **Hap42** (18); Hap73 (1)	0.00076	0.267 (0.120)
GZ	E1	Hap30 (3); Hap31 (1); **Hap42** (16)	0.00035	0.353 (0.123)
JJ	E1	hap11 (1); hap12 (1); Hap26 (1); Hap27 (1); Hap28 (1); Hap29 (1); **Hap36** (1); **Hap42** (15)	0.00069	0.545 (0.128)
CM	E1	Hap8 (1); Hap32 (1); Hap33 (1); Hap34 (1); Hap35 (1); **Hap36** (1); **Hap42** (14)	0.00067	0.521 (0.135)
NG	E1	Hap21 (1); **Hap42** (21)	0.00009	0.091 (0.081)
NT	E1	**Hap42** (11)	0	0
RD	E1	**Hap42** (6)	0	0
RW	E1	Hap37 (4); **Hap42** (20)	0.00028	0.290 (0.103)
XQ	E1	**Hap42** (24)	0	0
YB	E1	Hap13 (22); Hap14 (2); Hap74 (1); Hap75 (1)	0.00064	0.289 (0.115)
BJ	E1	**Hap42** (5)	0.00192	0.333 (0.215)
	E3	Hap53 (1)		
CD	E1	Hap17 (1); **Hap20** (3); **Hap42** (15)	0.00468	0.432 (0.126)
	W1	Hap65 (1)		
CN	E1	Hap18 (1); **Hap42 (2)**	0.00196	0.768 (0.080)
	E2	Hap46 (1); Hap47 (4); **Hap49** (9); Hap50 (1); Hap51 (2)		
NY	E1	**Hap42** (4)	0.00292	0.835 (0.062)
	E3	**Hap52** (1); Hap54 (1); Hap55 (4); Hap56 (3); Hap57 (1)		
QG	E1	Hap7 (1); Hap22 (1); Hap23 (2); Hap24 (1); Hap25 (1); **Hap42** (12)	0.00167	0.647 (0.120)
	E2	Hap44 (1); Hap45 (1)		
QS	E2	Hap48 (1); **Hap49** (19)	0.0001	0.100 (0.088)
SP	E1	Hap9 (6); Hap10 (3); **Hap42** (2)	0.00332	0.780 (0.085)
	E2	Hap43 (2)		
	E3	**Hap52** (1)		
LU	E4	Hap1 (6); Hap2 (4); Hap3 (1); Hap4 (1); Hap5 (8)	0.00094	0.742 (0.058)
LC	W1	Hap67 (1); **Hap68** (16)	0.00011	0.118 (0.101)
LL	W1	Hap59 (1); Hap62 (8); Hap63 (1); **Hap68** (10)	0.00079	0.616 (0.067)
LN	W1	**Hap68** (20)	0	0
PL	E1	**Hap20** (1)	0.00432	0.100 (0.088)

	W1	**Hap68** (19)		
RC	W1	Hap66 (1); **Hap68** (6)	0.00027	0.286 (0.196)
XH	W1	**Hap68** (2)	0	0
ZB	W1	Hap64 (1); **Hap68** (19)	0.00010	0.100 (0.088)
NM	W1	Hap58 (1); Hap60 (1); Hap61 (1); **Hap68** (3)	0.01109	0.892
	W2	Hap69 (6); Hap70 (1); Hap71 (1); Hap72 (1)		

π, nucleotide diversity; *h* (s.d.), haplotype diversity with standard deviation.

Table S3 Mean pairwise uncorrected *p*-distances (%) and pairwise Φ_{ST} values among Cyt*b* sublineages. Above the diagonal are the Φ_{ST} values. Pairwise *p*-distances between and within Cyt*b* lineages are shown below and at the diagonal, respectively.

	Cyt*b* sublineage	1	2	3	4	5	6
1	E1	0.24	0.83**	0.83**	0.91**	0.98**	0.98**
2	E2	0.62	0.23	0.69**	0.90**	0.98**	0.98**
3	E3	0.62	0.44	0.22	0.87**	0.97**	0.96**
4	E4	0.96	0.94	0.97	0.15	0.97**	0.97**
5	W1	4.35	4.29	4.26	4.29	0.25	0.94**
6	W2	4.33	4.27	4.24	4.27	1.96	0.32
7	outgroup	14.34	14.30	14.49	14.54	14.02	13.83

** denotes significance at $\alpha = 0.01$.

Table S4 PCR primer sets used in the study. Tm (℃) is the annealing temperature.

Locus	Abbreviation	Tm (℃)	Primer	Primer sequence	Reference
Mitochondrial cytochrome *b*	Cyt*b*	55	Cybt-F	5'-TCCAGCCCGAAAATCTCAC-3'	This study
			Cybt-R	5'-TGCACGTAACTGCCGCTAG-3'	
Cellular myelocytomatosis intron 2	*c-Myc2*	61	CYMC5	5'-ATCRTYATHCAGGACTGTATGTGGAG-3'	
			CYMC6	5'-GACCTSGGACTCGARCACTTGCG-3'	[1]
Rhodopsin	*Rhod*	60	Rhod1A	5'-ACCATGAACGGAACAGAAGGYCC-3'	[2]
			Rhod1D	5'-GTAGCGAAGAARCCTTCAAMGTA-3'	
Tyrosinase	*Tyr*	60	Tyr1G	5'-TGCTGGGCRTCTCTCCARTCCCA-3'	[2]
			Tyr1B	5'-AGGTCCTCYTRAGGAAGGAATG-3'	

1. Chen, W., Bi, K. & Fu, J. Frequent mitochondrial gene introgression among high elevation Tibetan megophryid frogs revealed by conflicting gene genealogies. *Mol. Ecol.* **18**, 2856-2876 (2009).

2. Bossuyt, F., Brown, R. M., Hillis, D. M., Cannatella, D. C. & Milinkovitch, M. C. Phylogeny and biogeography of a cosmopolitan frog radiation: Late Cretaceous diversification resulted in continent-scale endemism in the family Ranidae. *Syst. Biol.* **55**, 579-594 (2006).

Table S5 Occurrence data of *N. parkeri* for the species distribution modelling.

Species	Longitude	Latitude
Nanorana.parkeri	87.12483	28.43992
Nanorana.parkeri	86.0351	28.30842
Nanorana.parkeri	89.07698	29.56162
Nanorana.parkeri	90.88817	30.70533
Nanorana.parkeri	92.96918	31.86978
Nanorana.parkeri	96.5952	31.26215
Nanorana.parkeri	84.30112	29.64092
Nanorana.parkeri	86.52375	29.4737
Nanorana.parkeri	94.23038	31.86577
Nanorana.parkeri	91.1014	29.91587
Nanorana.parkeri	91.71733	29.83608
Nanorana.parkeri	91.07197	30.46238
Nanorana.parkeri	90.44918	30.05708
Nanorana.parkeri	94.34748	29.32328
Nanorana.parkeri	94.72995	29.70042
Nanorana.parkeri	93.64725	29.14377
Nanorana.parkeri	95.37265	31.4893
Nanorana.parkeri	97.2747	30.21913
Nanorana.parkeri	92.2555	29.6932
Nanorana.parkeri	91.92038	28.91742
Nanorana.parkeri	90.38728	29.00807
Nanorana.parkeri	89.1461	27.789
Nanorana.parkeri	89.59408	28.90305
Nanorana.parkeri	88.7134	29.35972
Nanorana.parkeri	87.68697	29.07023
Nanorana.parkeri	93.1917	29.89865
Nanorana.parkeri	92.355383	31.665217
Nanorana.parkeri	90.98333	29.55
Nanorana.parkeri	91.78852	31.29663
Nanorana.parkeri	91.964	28.004
Nanorana.parkeri	85.245	29.373
Nanorana.parkeri	88.97	27.53
Nanorana.parkeri	97.719	29.874
Nanorana.parkeri	85.338	28.324
